# A Network Meta-Analysis of the Dose–Response Effects of Dapagliflozin on Efficacy and Safety in Adults With Type 1 Diabetes

**DOI:** 10.3389/fendo.2022.923376

**Published:** 2022-07-07

**Authors:** Yinhui Li, Hui Li, Liming Dong, Dandan Lin, Lijuan Xu, Pengwei Lou, Deng Zang, Kai Wang, Li Ma

**Affiliations:** ^1^ Department of Endocrine, Traditional Chinese Medicine Hospital Affiliated to Xinjiang Medical University, Urumqi, China; ^2^ Department of Clinical Nutrition, The First Affiliated Hospital of Xinjiang Medical University, Urumqi, China; ^3^ College of Medical Engineering and Technology, Xinjiang Medical University, Urumqi, China; ^4^ Department of Medical Administration, Traditional Chinese Medicine Hospital Affiliated to Xinjiang Medical University, Urumqi, China

**Keywords:** dapagliflozin, type 1 diabetes, dosage, efficacy, safety

## Abstract

**Background:**

Most patients with type 1 diabetes (T1DM) do not reach the blood glucose goal with treatment of insulin. In our research, we intended to estimate the therapeutic effect and safety of additional different doses of dapagliflozin on insulin treatment in T1DM.

**Methods:**

We performed direct and indirect network meta-analysis using Bayesian models and graded different dosages of dapagliflozin by mixed therapy contrasts. We retrieved information from the PubMed, Embase, The Cochrane Library, Web of Science, China Biology Medicine (CBM) disc, China National Knowledge Infrastructure (CNKI), Wanfang Data, and WEIPU Data. Our research included randomized controlled trials (RCTs) including T1DM treated with insulin and additional dapagliflozin 5 mg or dapagliflozin 10 mg from January 2012 to June 2021. Thirteen RCTs with 10,701 participants were divided into three groups as below: insulin alone, dapagliflozin 5 mg + insulin, and dapagliflozin 10 mg + insulin.

**Results:**

Dapagliflozin dose-dependently exhibited reductions in glycated hemoglobin (HbA1c), total insulin daily dose (TDD), and body weight. Neither dapagliflozin 5 mg nor 10 mg could induce hypoglycemia or severe hypoglycemia. However, both doses of dapagliflozin increased the incidence of diabetic ketoacidosis (DKA) and genital infection.

**Conclusions:**

Dapagliflozin 10 mg could achieve a better outcome in efficacy and could not increase the risk of hypoglycemia. Although it may induce a higher risk of DKA and genital infection, there was no significant difference between dapagliflozin 10 mg and 5 mg. Our outcomes indicate that dapagliflozin 10mg has a high reliability of being graded prior as a supplementary treatment to insulin in T1DM.

## Introduction

The morbidity of type 1 diabetes (T1DM) is gradually increasing. According to the International Diabetes Federation (IDF), there are currently 29 million people worldwide who have T1DM (1). The IDF announced that 132,600 people have new-onset T1DM each year. T1DM is a dysfunction of autoimmunity attributed to definitely endogenic insulin absence; meanwhile, it is an accelerated metabolic disease that raises the risk of atherosclerotic disease and mortality (2, 3). Therefore, T1DM requires lifelong insulin substitute therapy with intense treatment to prevent macrovascular, microvascular, and other risks associated with hyperglycemia (4). Glucagon imbalances and the continual undulation in blood glucose are several restrictions of insulin treatment (5). Additionally, insulin therapy for a long time is unsatisfactorily associated with some disadvantages, such as insulin resistance, weight gain, dyslipidemia, and hypoglycemia (6, 7), that are of deep concern in T1DM because they could influence therapy compliance and result in an increased mortality rate (8, 9). Currently, the concerns of studies have been converted to the progress of innovative additional medicines that might aid in the administration of T1DM. The major objective is to povide superior glycemic control without increasing the rate of hypoglycemia and insulin dose.

Consequently, some medicines have been indagated as an additional treatment for T1DM, yet the Food and Drug Administration (FDA) only permitted pramlintide in 2005, which imitates a β-cell hormone that is co-secreted with insulin in the post-cibum time (10). Nevertheless, the effectiveness of pramlintide on glycosylated hemoglobin (HbA1c) and weight loss is moderate and unsatisfied (11) with some adverse reactions, including a higher risk of hypoglycemia and nausea (12). So it is necessary to look for new drugs to treat T1DM effectively. Sodium-glucose cotransporter-2 (SGLT-2) inhibitors, that decrease glucose reuptake in the proximal tubules of the nephridium, are one of the most dramatic medicines for type 2 diabetes (T2DM) because of their advantages on renal and cardiovascular functions (13). A research in mouse models of DM indicated that SGLT2 inhibitors could lead to β-cell regeneration by decreasing glucose-induced β-cell toxicity and ameliorating pancreatic β-cell function. They preserved islet mass, where the frequency of cell death significantly decreased (14). Therefore, the SGLT-2 inhibitor is an appropriate therapy for T1DM. Previous reports showed that, in contrast with insulin therapy alone, adding SGLT-2 inhibitors significantly reduced HbA1c, insulin dose, and body weight in T1DM (15, 16). Dapagliflozin, the first and representative drug of SGLT-2 inhibitor, has been recently researched popularly for the hyperglycemic effect and side-reactions for treating T1DM (17). How to equilibrate the curative effect and adverse reactions, particularly in the field of dosage, presents a prominent issue in clinical practice. Several trials with dapagliflozin as an addition to insulin treatment for T1DM have been going on, whereas, no definite advice has been concluded for the reasons as follows. The hypoglycemic effect and safety for different dosages of dapagliflozin were incomplete. These studies had a small sample size and evaluated partial outcomes. Therefore, our network meta-analysis tried to estimate the dosage-dependent efficacy of dapagliflozin versus placebo in association with insulin treatment for glycemic level as measured by the variances in HbA1c, total insulin daily dose (TDD), and the change of 24 h continuous glucose monitoring value (24 h CGM value). Body weight and adverse events were also analyzed. We aimed to perform a network meta-analysis to find the most effective and safe dose of dapagliflozin as an adjunct to insulin treatment in T1DM.

## Methods

### Data Sources and Search Strategy

The databases below were searched: PubMed, Embase, The Cochrane Library, Web of Science, China Biology Medicine (CBM) disc, China National Knowledge Infrastructure (CNKI), Wanfang Data, and WEIPU Data from January 2012 till 3 June 2021. The databases were searched by a combination of subject search and free search, and the randomized controlled trials (RCTs) followed the search strategy provided by the Cochrane Collaboration. The corresponding search term combinations were (“dapaglipflozin”) AND (“Diabtetes Mellitus, Type 1”) AND (“randomized”). There was a language restriction, and we used results from Chinese and English-language literature. The search strategy is shown in **Appendix 1**. The outcomes were exhibited according to the guidelines of the Preferred Reporting Items for Systematic Reviews and Network Meta-Analyses statement (NMA Checklist) (18). Total analyses were managed using previously published studies, so approval of ethics and consent of the patient were not needed.

### Inclusion and Exclusion Criteria

The criteria for inclusion for studies included: (1) randomized controlled studies (RCTs); (2) studies relating to at least two of the eligible antidiabetic medicines below: placebo combined with insulin; dapagliflozin 5 mg combined with insulin; and dapagliflozin 10 mg combined with insulin; (3) studies that contained at least one primary or secondary outcome; and (4) patients with T1DM aged above 18 years old. Studies were excluded as follows: (1) Studies that recruited patients with diabetes mellitus of other types, including T2DM or latent autoimmune diabetes, etc. (2) Studies that were on non-RCTs, non-clinical patients, case reports, letters, as well as studies that did not contain interesting results or required indexes.

**Figure 1:**
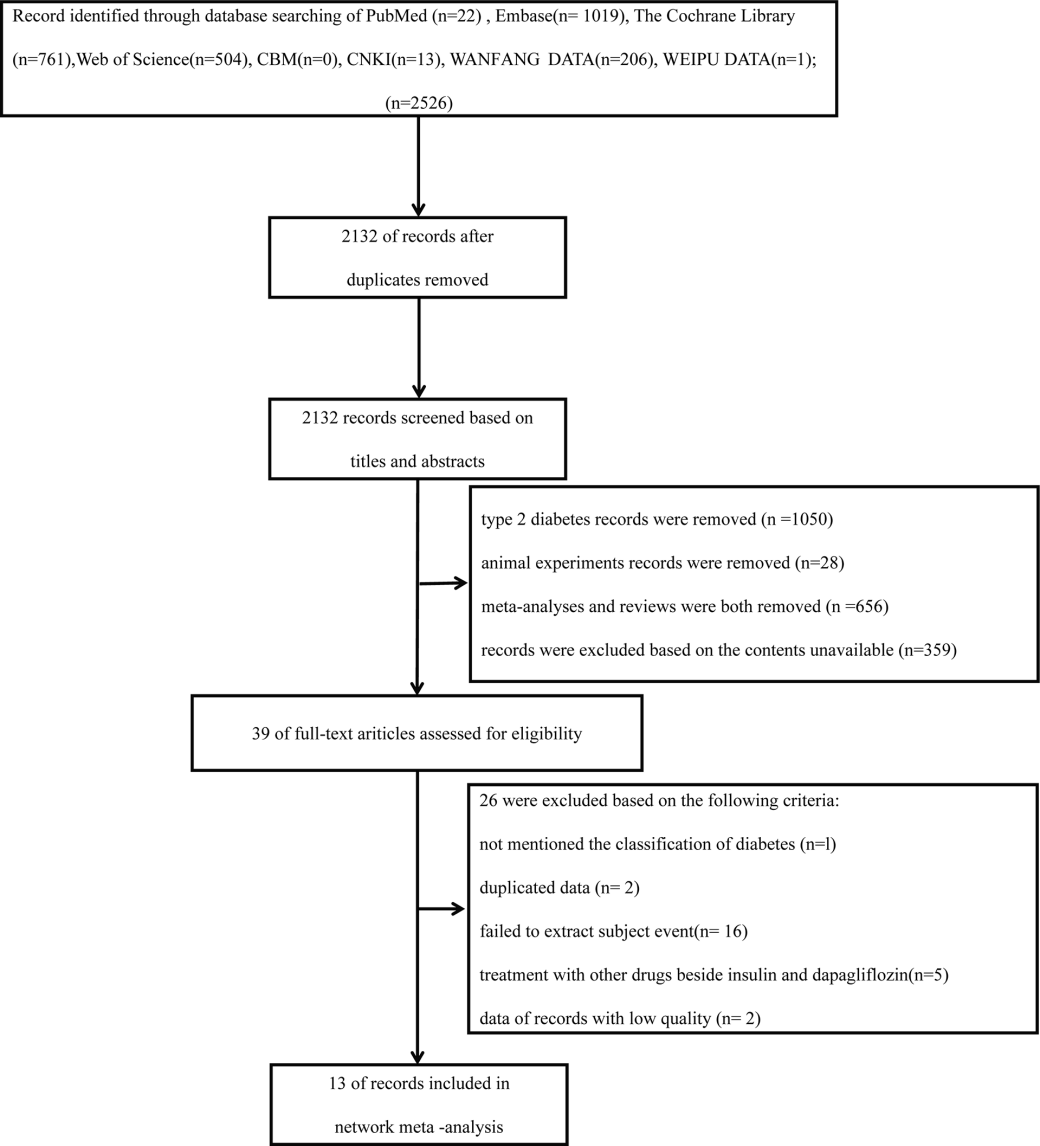
Flow graph of the present systematic review (PRISMA Flow Diagram).

These studies below were included in the articles: reviews, RCTs, clinical trials, and observational studies. The search was restricted to human research and was limited to Chinese and English. Reference lists from all accessible literature and RCTs were manually searched (**Figure 1**).

### Study Selection and Data Extraction

The outcomes of the systematic searches were input into NoteExpress software (3.4.0.8878), a reference manager. Two reviewers (LD and HL) filtered topics, abstracts, and full-text literatures independently. Any inconsistencies were settled by discussions and consultations with the third researcher (LX). We intended to confirm the effect of dapagliflozin on variance in HbA1c as the primary result. The reduction in TDD, the change in 24 h CGM value, the change in body weight, and the occurrence of diabetic ketoacidosis (DKA), urinary tract infection, genital infection, hypoglycemia, and severe hypoglycemia were determined as secondary outcomes. The extraction included: for every study, total information originating from the published texts or tables was tabulated into a Microsoft Excel sheet and the matching research characters were extracted. The whole information came from every experiment. We involved the number of patients with T1DM from the baseline, dosage of medicines (dapagliflozin 5 mg, dapagliflozin 10 mg, and placebo with basic treatment of insulin), follow-up duration, and key study information (primary and secondary outcomes).

### Quality Assessment

**Figure 2:**
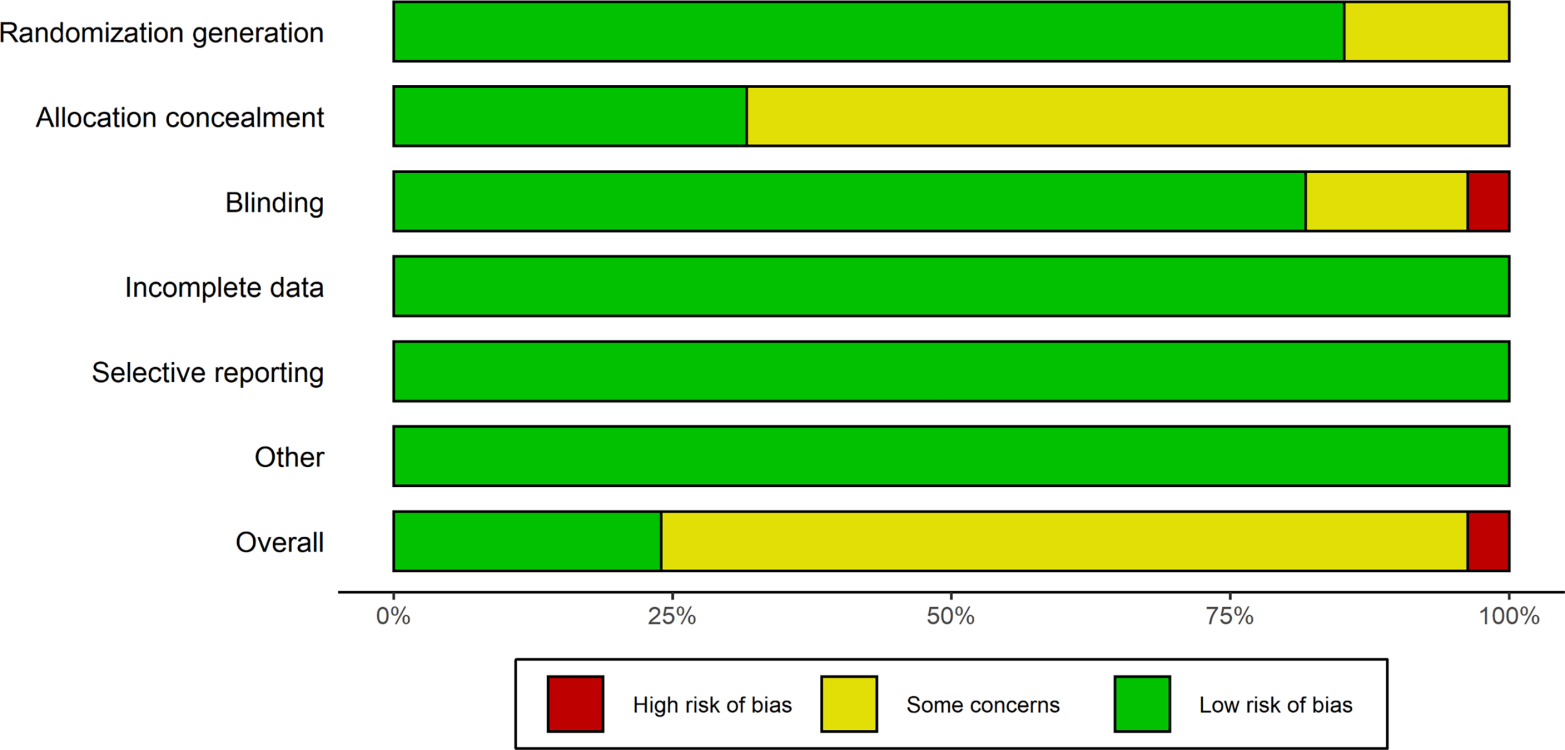
Risk of bias diagram. Evaluation of every risk of bias item is shown as a cumulative percentage of all.

**Figure 3:**
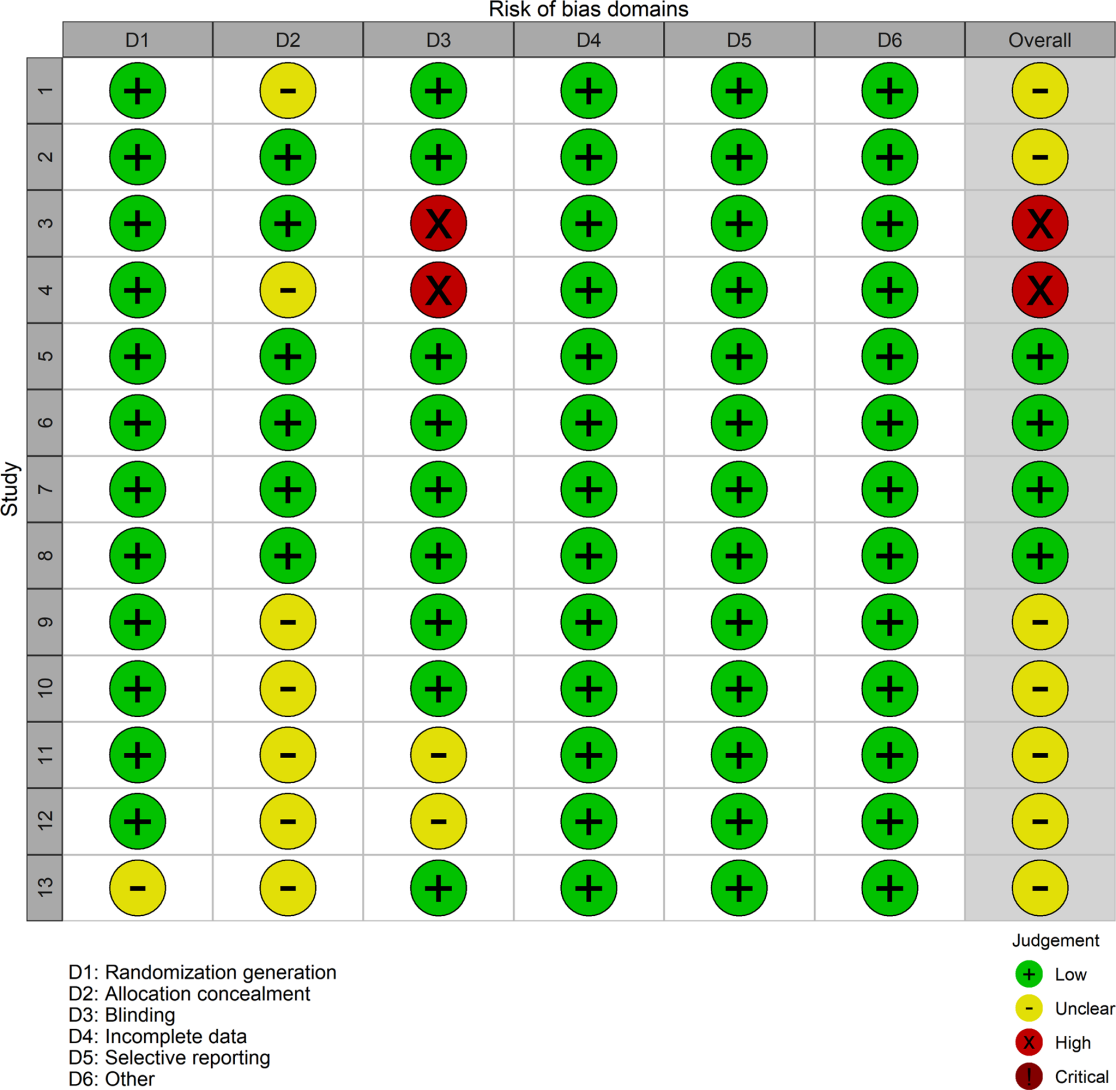
Risk of bias diagram and summary evaluation of every risk of bias item for each involving experiment. Plus green circle, good; yellow circle, moderate; minus red circle, bad.

The risk of bias evaluation iss exhibited in **Figures 2**, **3**. The researchers (HL and LD) estimated the risk of bias of every experiment using the Cochrane Collaboration’s Risk of Bias tool independently (19). The risk of bias was evaluated during the generation of a random list, concealment of allocation, blinding of participants and personnel, blinding of result evaluation, analysis of incomplete data, selective reporting, and others. All the judgments were classified as “yes” (low risk of bias) or “unclear” (indicating that the literature provides insufficient or uncertain information for assessing bias) or “no” (high risk of bias) (20). Although most involved studies were described as randomized with allocation concealment, a few studies did not provide definite details of either the method of randomization or concealment of allocation. As well as some involving studies, blinding had not been carried out sufficiently.

### Data Synthesis and Statistical Analysis

A Bayesian network meta-analysis was conducted to compare the effects of three hypoglycemic therapies in the fields of HbA1c change, reduction of TDD, change of 24 h CGM value, weight loss outcome, and adverse outcomes in patients with T1DM. We used insulin as a reference treatment in all analyses. Direct and indirect network meta-analyses were conducted using Bayesian models, and the various agents were graded through mixed therapy contrast and using R software version 4.0.1 (R foundation; packages meta, gemtc, coda, pcnetmeta, and rjags) (21, 22). The related ranking possibility of every therapy was evaluated, and the therapy hierarchy of contesting interferences was gained using rankograms and mean ranks. The network meta-analysis was conducted on experiments estimating multiple therapies; this allowed the evaluation of pooled efficacy within every therapy (23). For multi-arm studies, correlations among the therapy efficacy among arms were included in the surveys. Trials with j + 1 therapy arms were on account of the contrast of the therapy efficacy with the reference therapy efficacy according to multivariate normal distribution, while therapy-as-usual trials were on account of the homogeneity among trail changes across therapies (24). Inconsistency tests, homogeneity analysis, and sensitivity analysis were conducted utilizing the node analysis method in R software. The outcomes of inconsistency tests were evaluated through the Bayesian p-value, where the results with *p <*0.5 were believed to be significant inconsistencies (25). An I^2^ test was conducted (I^2^ >50% demonstrated significant heterogeneity) to assess homogeneity. Moreover, a sensitivity analysis was performed by comparing the differences between fixed-effect and random-effects models. The clinical indexes were assessed using the mean difference or odds ratio (OR) with a 95% confidence interval (CI) (mean difference for continuous outcomes and OR for binary outcomes) (26). When a loop linked three therapies, it was possible to estimate the inconsistency between direct and indirect proof. We also utilized the node-splitting method to figure out the inconsistency of the model, which divided proof for a special comparison into direct and indirect proof. Thus, the consistency between direct and indirect proof was estimated, and its Bayesian *p*-value was achieved.

## Results

### Identified Publications

In total, 2,526 articles were preliminarily reviewed from the electronic database search. A total of 394 duplicate articles were eliminated. After reading the titles and abstracts, 1,050 T2DM articles were removed; 28 animal experiments were removed; 536 meta-analyses and 120 reviews were removed. A total of 359 were excluded based on the contents being unavailable. Thirty-nine articles have been retrieved for full-text review. Among these articles, 26 were excluded on account of the criteria below: not mentioning the classification of diabetes (n = 1), duplicated data (n = 2), failed to extract subject event (n = 16), treatment with other drugs besides insulin and dapagliflozin (n = 5), data of records with low quality (n = 2) (**Figure 1**). Ultimately, 13 trials covering results for 10,701 patients (5,669 women and 5,032 men) were involved in the analysis. The mean study duration was 41.54 ± 14.47 weeks (**Supplementary Materials Table S1**) (15, 16, 27-37).

The risk of bias evaluation is shown in **Figures 2**, **3**. Although most involved articles were depicted as randomization and allocation concealment, a few articles did not provide particular details on either the method of randomization or concealment of allocation. For some involving articles, blinding was not done sufficiently.

**Figure 4:**
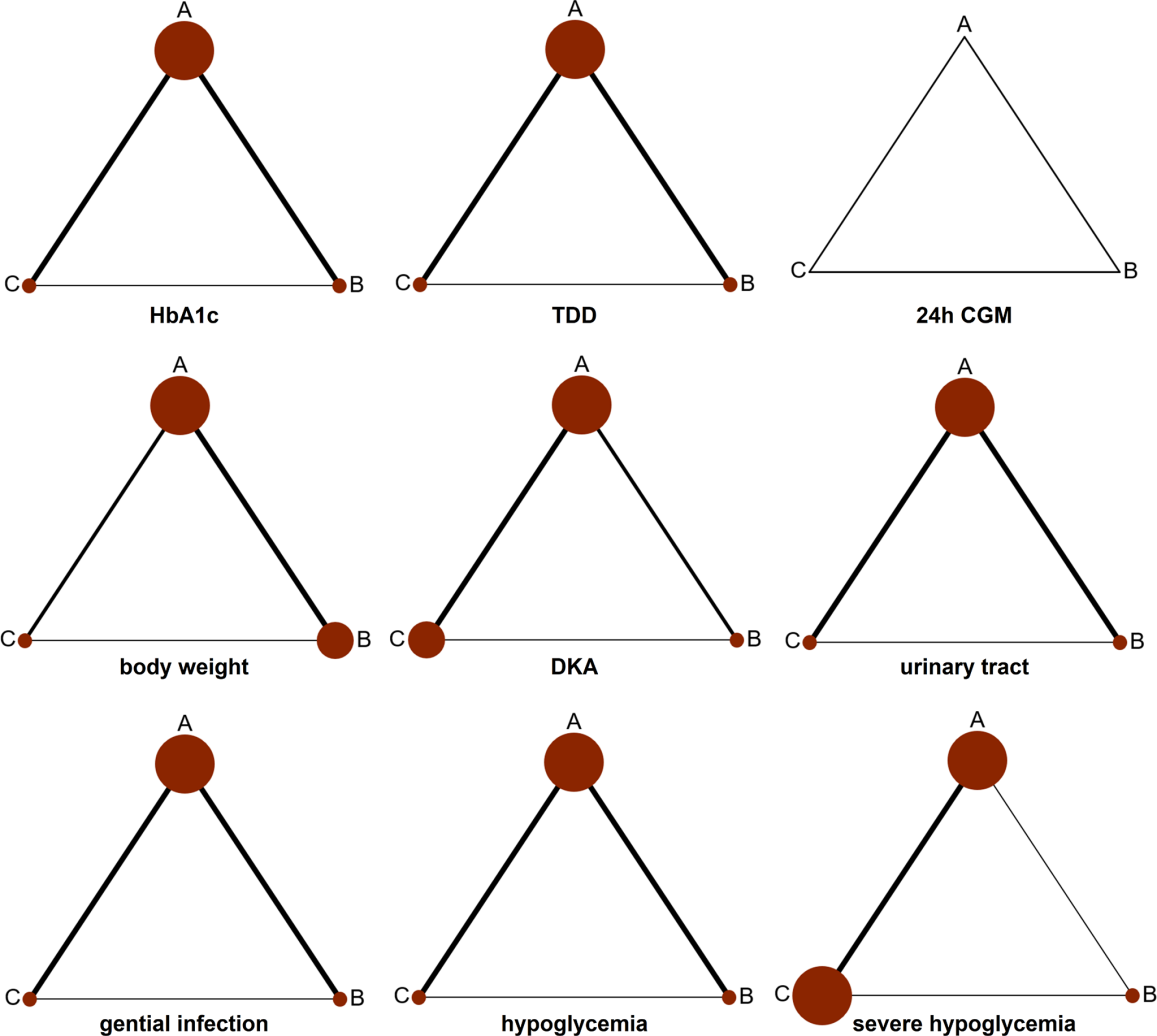
Network diagram for analyzing the efficacy and safety of categorized 3 different interventions. The width of the lines presents the whole number of trials for every comparison, and the size of every node is proportional to the number of randomized participants (sample size). (A) dapagliflozin 5 mg + insulin, (B) dapagliflozin 10 mg + insulin, and (C) placebo + insulin.

Data acquired from the whole 13 articles (n = 10,701) were presented to the network analysis. All involved RCTs compared dapagliflozin with a placebo on the basis of insulin therapy. While published articles provided results for different follow-up periods, the primary and secondary results from each follow-up period were employed for effect analysis. Thirteen sub-studies of HbA1c were designed to compare each other with dapagliflozin 5 mg, dapagliflozin 10 mg, and insulin alone. Ten sub-trials researching TDD were designed to compare each other among the three groups. Seven sub-trials about 24 h CGM were aimed to compare each other among the three groups. Sixteen sub-trials about weight loss were devised to compare each other among the three groups. Thirteen sub-trials about DKA were designed to compare each other among the three groups. Nine sub-trials about urinary tract infection were designed to compare to each other among the three groups. Eight sub-trials about genital infection were designed to compare each other among the three groups. Twelve sub-trials about hypoglycemia were designed to compare each other among the three groups. Nine sub-trials of severe hypoglycemia were designed to compare each other among the three groups. The network of eligible comparisons is presented in **Figure 4**.

### Network Meta-Analysis Results

#### HbA1c

**Figure 5:**
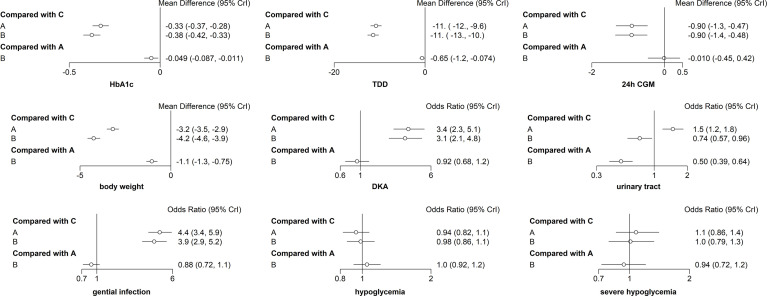
Forest map meta-analysis of different interventions and outcome indicators. (A) dapagliflozin 5 mg + insulin, (B) dapagliflozin 10 mg + insulin, and (C) placebo + insulin.

**Figure 6:**
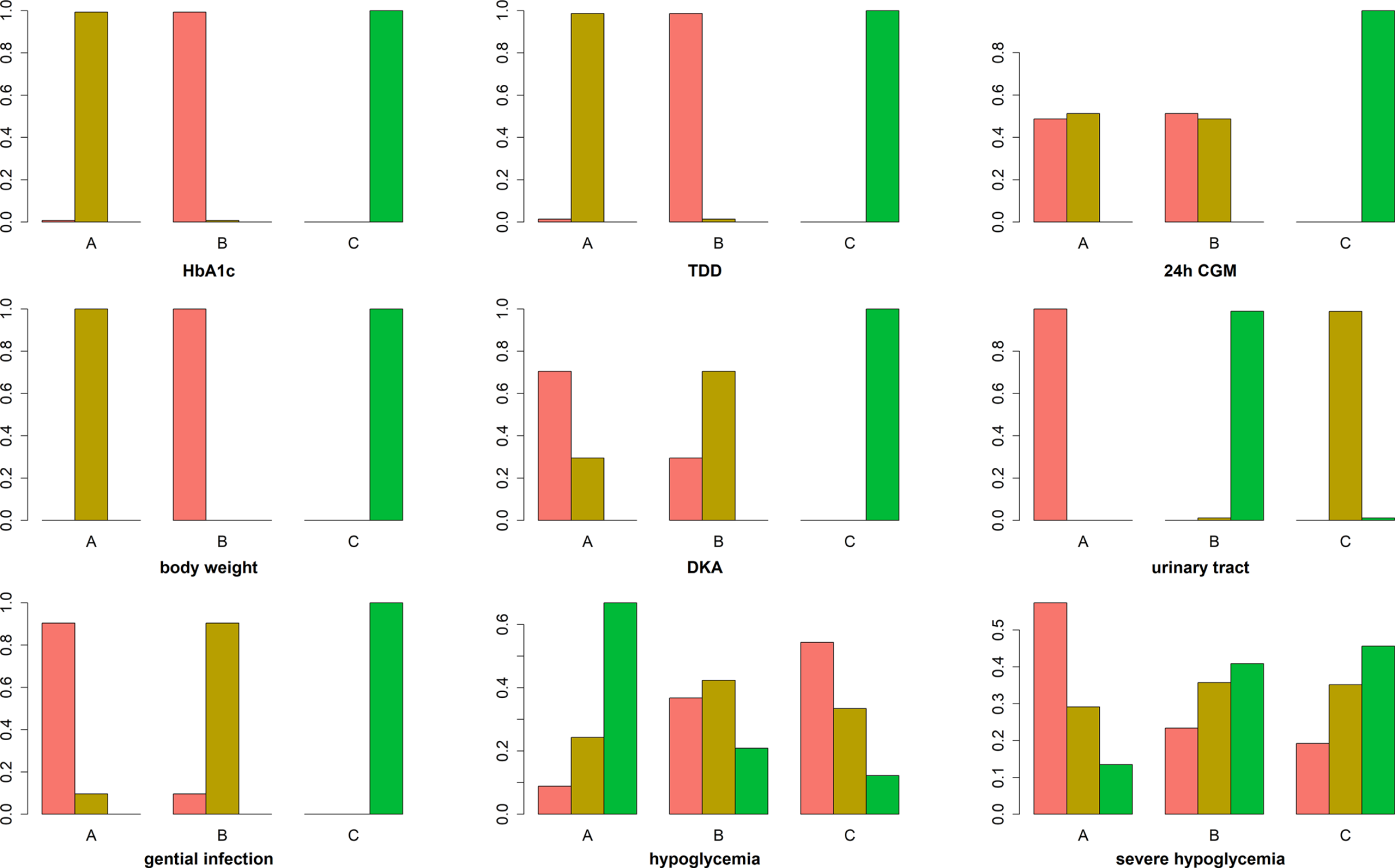
Ranking chart of meta-analyses of different interventions and outcomes. The width of the lines presents the whole number of trials for every comparison, and the size of every node is proportional to the number of randomized participants (sample size). (A) dapagliflozin 5 mg + insulin, (B) dapagliflozin 10 mg + insulin, and (C) placebo + insulin.

The primary outcome included changes in HbA1c. Compared with the insulin alone therapy as the reference, dapagliflozin 5mg therapy significantly decreased the HbA1c level (MD: −0.33, 95% CI: −0.37 to −0.28), dapagliflozin 10mg therapy significantly decreased the HbA1c level (MD: −0.38, 95% CI: −0.42 to −0.33). Compared with dapagliflozin 5 mg, dapagliflozin 10 mg therapy significantly decreased the HbA1c level (MD: −0.049, 95% CI: −0.087 to −0.011). (**Figure 5**). **Table 1** and **Figure 6** show that dapagliflozin 10 mg might be the most efficient at reducing HbA1c levels in T1DM. The particular outcomes of the comparison of the efficacy of 5 mg, 10 mg of dapagliflozin, and placebo on HbA1c levels are presented in the **Supplementary Materials** (**Table S2**).

**Table 1:** SUCRA values of different interventions and outcomes were analyzed.

	HbA1c	TDD	24 h CGM	Body weight	DKA	Urinary tract	Genital infection	Hypoglycemia	Severe hypoglycemia
A	0.50	0.51	0.74	0.5	0.84	1	0.93	0.11	0.71
B	0.99	1	0.76	1	0.66	0.01	0.57	0.69	0.40
C	0	0	0	0	0	0.49	0	0.70	0.38

A, dapagliflozin 5 mg + insulin; B, dapagliflozin 10 mg + insulin; C, placebo + insulin.

#### Secondary Endpoints

We further analyzed TDD, 24 h CGM value, weight variance, and side effects as the secondary outcomes.

##### TDD

Among the ten sub-study agents, dapagliflozin 5 mg reduced the change in TDD compared to insulin alone (MD: −11%, 95% CI: −12 to −9.6%), dapagliflozin 10 mg decreased the change in TDD compared with insulin alone (MD: −11%, 95% CI: −13 to −10%), and dapagliflozin 10 mg decreased the change in TDD compared with 5 mg dapagliflozin (MD: −0.65%, 95% CI: −1.2 to −0.074%) (**Figure 5**). **Table 1** and **Figure 6** show that dapagliflozin 10 mg might be the most efficient at reducing TDD levels in T1DM. The particular outcomes of the comparison of the efficacy of 5 mg, 10 mg of dapagliflozin, and placebo on TDD levels are exhibited in the **Supplementary Materials** (**Table S3**).

##### 24h CGM

The 24-h CGM value represents the mean value of 24-hour glucose readings obtained from continuous glucose monitoring. It could make up for the shortage of HbA1c, which reflects overall glucose exposure over time, without assessing glycemic variability. 24-h CGM enables detection throughout the day of significant glycemic variables. The change from baseline in 24 h CGM value showed significant improvements for both dapagliflozin doses versus insulin alone. The mean change from baseline in 24 h CGM value versus insulin alone was −0.90 mmol/l (95% CI: −1.3 to −0.47) and −0.90 mmol/l (95% CI: −1.4 to −0.48) for dapagliflozin 5 and 10 mg, respectively. The decrease in 24 h CGM value from baseline in the dapagliflozin 10 mg was not significant different from that in the dapagliflozin 5 mg (−0.010 mmol/l, 95% CI: &minus;0.45 to 0.42) (**Figure 5**). **Table 1** and **Figure 6** show that dapagliflozin 10 mg might be the most efficient at reducing the 24 h CGM value in T1DM. The particular outcomes of the comparison of the efficacy of 5 mg, 10 mg of dapagliflozin, and placebo on the 24 h CGM value are shown in the **Supplementary Materials** (**Table S4**).

##### Body Weight

Compared with insulin alone, dapagliflozin 5 mg decreased the percentage of body weight from the baseline significantly (MD: −3.2%, 95% CI: −3.5 to −2.9%). Another reduction in body weight percentage from the baseline was also discovered after therapy with dapagliflozin 10 mg compared with insulin alone (−4.2%, 95% CI: −4.6 to −3.9%). Compared with dapagliflozin 5 mg, dapagliflozin 10 mg decreased body weight percentage from the baseline significantly (−1.1%, 95% CI: −1.3 to −0.75%). (**Figure 5**). **Table 1** and **Figure 6** show that dapagliflozin 10 mg might be the most efficient at reducing body weight level in T1DM. The particular outcomes of the comparison of the efficacy of 5 mg, 10 mg of dapagliflozin, and placebo on body weight levels are exhibited in the **Supplementary Materials** (**Table S5**).

##### DKA

DKA is one of the most severe side reactions discovered in T1DM. In our study, compared with insulin alone, DKA was more frequently observed with both dapagliflozin 5 and 10 mg (OR = 3.4, 95% CI: 2.3 to 5.1 and OR = 3.1, 95% CI: 2.1 to 4.8) treatments. The rate of DKA in dapagliflozin 10 mg was not different from that in dapagliflozin 5 mg (OR = 0.92, 95% CI: 0.68 to 1.2) (**Figure 5**). **Table 1** and **Figure 6** show that dapagliflozin 5 mg may be most likely to increase the rate of DKA in patients with T1DM. The particular outcomes of the comparison of the efficacy of 5 mg, 10 mg of dapagliflozin, and the placebo on the rate of DKA are shown in the **Supplementary Materials** (**Table S6**).

##### Urinary Tract Infection

Urinary tract infection is a common adverse effect in T1DM when using dapagliflozin. Compared with insulin alone, dapagliflozin 5 mg increased the occurrence of urinary tract infection (OR = 1.5, 95% CI: 1.2 to 1.8). There was a slightly significant difference in the occurrence of urinary tract infection between dapagliflozin 10 mg and insulin alone (OR = 0.74, 95% CI: 0.57 to 0.96). Dapagliflozin 10 mg decreased the occurrence of urinary infection significantly more than dapagliflozin 5 mg (OR = 0.50, 95% CI: 0.39 to 0.64) (**Figure 5**). **Table 1** and **Figure 6** show that dapagliflozin 5 mg may be most likely to increase the occurrence of urinary infection in patients with T1DM. The particular outcomes of the comparison of the efficacy of 5 mg, 10 mg of dapagliflozin, and placebo on the occurrence of urinary infection are shown in the **Supplementary Materials** (**Table S7**).

##### Genital Infection

For the side effects of particular interest, there were more genital infections in the dapagliflozin therapy group than in the insulin alone group. Both dapagliflozin 5 and 10 mg increased the risk of genital infection versus insulin alone (OR = 4.4, 95% CI: 3.4 to 5.9 and OR = 3.9, 95% CI: 2.9 to 5.2). There was no significant difference in the risk of genital infection between dapagliflozin 5 and 10 mg (OR = 0.88, 95% CI: 0.72 to 1.1) (**Figure 5**). **Table 1** and **Figure 6** show that dapagliflozin 5 mg may be most likely to raise the risk of genital infection in T1DM. The particular outcomes of the comparison of the efficacy of 5 mg, 10 mg of dapagliflozin, and the placebo on the risk of genital infection are shown in the **Supplementary Materials** (**Table S8**).

##### Hypoglycemia and Severe Hypoglycemia

In the study, dapagliflozin 5 and 10 mg did not raise the incidence of hypoglycemia compared with insulin alone (OR = 0.94, 95% CI: 0.82 to 1.1 and OR = 0.98, 95% CI: 0.86 to 1.1). There was no difference in the incidence of hypoglycemia between dapagliflozin 10 mg and dapagliflozin 5 mg (OR = 1, 95% CI: 0.92–1.2) (**Figure 5**). Compared with insulin alone, dapagliflozin 5 and 10 mg did not increase the incidence of severe hypoglycemia (OR = 1.1, 95% CI: 0.86 to 1.4 and OR = 1, 95% CI: 0.79 to 1.3). The incidence of severe hypoglycemia in dapagliflozin 10 mg was not different from that in dapagliflozin 5 mg (OR = 0.94, 95% CI: 0.72 to 1.2) (**Figure 5**). **Table 1** and **Figure 6** show that both dapagliflozin 5 and 10 mg are unlikely to increase the incidence of hypoglycemia in patients with T1DM. Dapagliflozin 5 mg may be raising the incidence of severe hypoglycemia in T1DM. The particular outcomes of the comparison of the efficacy of 5 mg, 10 mg of dapagliflozin, and placebo on the incidence of hypoglycemia and incidence of severe hypoglycemia are shown in the **Supplementary Materials** (**Table S9 and Table S10**).

### Network Heterogeneity and Inconsistency

We separately built fixed and random effects models for all outcome indicators, which were used to select appropriate models for network meta-analysis. The results showed that for HbA1c, 24 h CGM, body weight, and severe hypoglycemia, the I^2^ of the fixed-effects model was larger than that of the random-effects model. A fixed-effects model should be used for network meta-analysis for these indicators. Simultaneously, for the remaining outcome indicators, the I^2^ of the random-effects model was larger than that of the fixed-effects model. A network meta-analysis should be performed using a random-effects model for these indicators (**Supplementary Data Table S11**). The assessment of global heterogeneity found I^2^ <30% for all different outcomes except for 24 h CGM, indicating low global heterogeneity (**Supplementary Data Table S12**). However, 24 h CGM had no I^2^ value because the heterogeneity test could not be performed. **Supplementary data** showed that, except for 24 h CGM, hypoglycemia, and severe hypoglycemia, the *P*-values of the discordance test for other outcome indicators were all greater than 0.05, and there was no local discordance (**Supplementary Data Table S13**). For hypoglycemia, there was discordance between placebo + insulin vs dapagliflozin + insulin (*p <*0.05). For 24 h CGM and severe hypoglycemia, there was no comparison to assess inconsistency. The trace map, density map, and convergence diagnostic diagram for all outcome indicators are shown in the **Supplementary Material** (**Figures S1–S9**).

## Discussion

Owing to the complications of therapy, hypoglycemia, and the probability of weight increase, attaining and keeping the goal level of HbA1c by insulin optimization strategies remains a primary problem for T1DM. Even though development has been done in insulin preliminarily, medical delivery systems and glycemic monitoring, no more than one-third of patients are capable of achieving the glycemic goal, but considerable patients gain weight or are obese (38). The mechanism of dapagliflozin is to promote glycemic disposal in an insulin-independent way, thereby decreasing postprandial glycemia and glycemic excursions accompanied by an inferior insulin demand without increasing the risk of hypoglycemia. The outcomes of the DEPICT-1 and DEPICT-2 studies urged the European Medicines Agency (EMA), the National Institute of Healthcare and Excellence (NICE) in the United Kingdom, and the Ministry of Health, Labour and Welfare of Japan to authorize dapagliflozin as an additional medicine for T1DM and BMI of ≥27 kg/m^2^ (39). Our research found that dapagliflozin exhibited reductions in HbA1c, TDD, 24h CGM, and body weight.

It was previously demonstrated that SGLT2 inhibitors as additions to insulin in T1DM treatment showed a great decrease in glucose level, body weight, and insulin dosage (3, 40–42). So did dapagliflozin. One of the major advantages of dapagliflozin as an addition is the decrease in insulin burden in a pancreas-independent way. Reductions in body weight are considered to be acquired by glucosuria, osmotic diuresis, and calorie loss (43). Furthermore, the natriuretic nature of this medicine might further supply potential renal and cardiovascular benefits (44). However, due to a few dosage subgroup analyses, dosage-dependent efficacies for most indicators were not paid enough attention to by prior meta-analyses. Li et al. found that HbA1c and body weight were deeply reduced by a high dosage rather than a moderate dosage of dapagliflozin. However, in other indexes associated with glucose level, both high and moderate dosages were clinically significant without any dosage-directed difference between each other (45). Our analysis also noticed that dapagliflozin 10 mg added on insulin may have more beneficial efficacy in reducing HbA1c and body weight than dapagliflozin 5 mg added on insulin and insulin alone in patients with T1DM. Moreover, we found that the decrease in TDD was positively proportional to the doses of dapagliflozin. Because dapagliflozin reduced total insulin dosage, hypoglycemia due to high insulin dosage was impossible to occur theoretically. In our study, combination treatment of dapagliflozin and insulin did not raise the risk of hypoglycemia, and the same outcome was also obtained for severe hypoglycemia. This is identical with other meta-analysis (3, 40, 41). AEs including DKA, urinary infection, and genital mycotic infection were caused by high and moderate dosages of dapagliflozin (45). Our study also found both dapagliflozin 5 and 10 mg increased the risk of DKA and genital infection, but we made an interesting discovery. Dapagliflozin 5 mg add-on insulin increased the occurrence of urinary tract infection more than placebo add-on insulin. However, dapagliflozin 5 mg significantly increased occurrence of urinary infection significantly than dapagliflozin 10 mg. The increase in the number of urinary tract infections that is usually observed with SGLT2 inhibitors was also seen with dapagliflozin 5 mg in studies in T1DM. The ratio of patients reporting these infections was similar in this subgroup to that reported in the total populations of DEPICT-1 and -2 studies (15, 16, 27, 32). The occurrence of urinary tract infection was balanced across treatment groups but was more common in women than in men. We looked into the original studies, and found the proportion of female in dapagliflozin 5 mg group was higher than that of the dapagliflozin 10 mg group. This could be a major bias to confound the outcome. However our study found that there was no gender classification of urinary tract infection because the results of the included studies did not provide this. Further studies should evaluate the occurrence of urinary tract infection for different dosages of dapagliflozin in T1DM according to gender classification. On the other hand, we guess patients who take dapagliflozin may drink more water to avoid urinary tract infections, especially at the high dose, since the incidence of urinary tract infections was lower in dapagliflozin 10 mg than in 5 mg. Disposal of glucose according to urine excretion could result in a rising rate of genital infection, so both dapagliflozin 10 mg and 5 mg also increased the risk of genital infection. For the increasing occurrence of DKA by dapagliflozin, a relevant reason includes that dapagliflozin could raise the accumulations of acetoacetate and β-hydroxybutyrate in blood and urine. Furthermore, as is the reason for the hypoglycemic efficacy of dapagliflozin, the dose of insulin should have been decreased and resulted in lowering levels of antilipolytic activity and promotion of the production of free fatty acids. Thenceforth, the free fatty acids can shift to ketone bodies by the process of β-oxidation, which happens in the liver, reduce the elimination and increase the reabsorption of ketone bodies by the kidneys, leading to the consequence of DKA in some T1DM (46). Other researchers found most cases of DKA were induced or driven by missed insulin dosage, failure of the insulin pump, or alcohol uptake. Furthermore, almost all incidences were not severe and effectively dealt with conservative methods (15, 47). Although the problem-free resolution of the incidences announced in the experiments, DKA could usually result in destructive effects. Thereafter, cautions among patients, doctors, and caregivers are needed, such as avoiding inducing factor (e.g., failure of insulin pump and missed insulin dosage). Patients ought to be compliant with stringent glycemia and ketone monitoring. The rate of routine monitoring ought to be individual on account of patient-particular risk factors. To direct prophase discovery of euglycemic DKA, patients ought to be taught about DKA symptoms (fatigue, nausea, vomiting, loss of appetite, malaise, dyspnea, and weakness) (48). If the ketone level of the patient is increasing or DKA symptoms happen, the medicine ought to be suspended promptly and other medical care should be performed. Though DKA have undesirable efficacies for the patients, current beneficial efficacies of ketone body generation and its transform in fuel energetics have been indicated on account of the discoveries of cardiovascular benefits related with dapagliflozin (49).

Our research had some strengths. A traditional meta-analysis can only compare two groups on account of one intervention; it is just based on the homogeneity among study variances across therapies. That is a limitation. But network meta-analysis is a way to supplement interventions, groups, or conflict favors that are too puzzling to be directly compared with each other. Theoretically, network meta-analysis could be described as a statistical compound of all suitable evidence for an outcome from some studies across multiple therapies to create estimates of pairwise comparison of each intervention to every other intervention within a network. In our research, we conducted a direct and indirect analysis on the computer and graded among interventions by comparing three groups simultaneously using network meta-analysis. It can be helpful in selecting a different dosage of dapagliflozin add-on insulin for a specific condition. Another advantage of this analysis is that it constitutes a meantime analysis of all possible treatment choices and makes full use of the appropriate evidence within a single analysis. Therefore, it provides a more precise assessment of the clinical perspective and enables us to make a better decision. Traditional meta-analysis was limited by a few trials with direct comparisons between two therapies. Instead, network meta-analysis could be performed if both therapies have been compared to a common comparator, such as adding dapagliflozin 5 or 10 mg to insulin compared to insulin alone. Meanwhile, the relative ranking probability of each treatment was estimated, and the treatment hierarchy of competing interventions was obtained using rankograms and mean ranks. Finally, we solely involved well-designed RCTs. Thereafter, less accurate studies were removed, and the outcomes were less biased to raise reliability. Until now, there has not been a meta-analysis with a larger sample size than ours, as well as combining with two-rank dosage subgroup analysis for dapagliflozin in the treatment of T1DM. We also supplied more specific details on dose-associated effects and safety.

Our research also had some limitations. Several studies were conducted for a short time. Hence, the evaluation of long-term results, including renal complications and cardiovascular events, was not conducted. The second limitation was that some trials were incomplete and we did not gain the baseline characteristics. Third, when published studies reported results for different follow-up periods, we used each result from a different follow-up period for analysis. Therefore, some trials were divided into many subgroups, this may increase the bias of the result by our analyses. Finally, the usability of outcomes in the real-world could be indeterminacy because total involving studies were RCTs, where strict criteria with rigorous monitoring of care and fulfilling during every procedure application, and participants are possible to be treated positively.

In conclusion, dapagliflozin 10 mg treatment greatly decreased the HbA1c, insulin dosage, and body weight without increasing the risk of hypoglycemia in T1DM. But it may cause a higher risk of DKA and genital infection. There was no significant difference between dapagliflozin 10 mg and 5 mg. Our results indicate that dapagliflozin 10 mg may be a better choice as an additional treatment to insulin in T1DM. The beneficial efficacy of dapagliflozin 10 mg could be particularly significant for patients who fail to attain ideal glycemic goals for insulin alone. Meanwhile, side reactions related to dapagliflozin should be noted in patients. The correlation between drug dosage and safety still requires large-scale clinical trials for further exploration and research in the future.

## Data Availability Statement

The original contributions presented in the study are included in the article/**Supplementary Material**. Further inquiries can be directed to the corresponding authors.

## Author Contributions

Conceptualization: YL and LM. Data extraction: YL, LD, and LX. Formal analysis: DL and KW. Funding acquisition: KW. Investigation: YL, LM, and KW. Methodology: DL and KW. Software: DL and KW. Validation: PL and DZ. Writing-original draft: YL, DL, and LD. Writing-review and editing: KW and LM. All authors listed have made a substantial, direct, and intellectual contribution to the work and approved it for publication. All authors contributed to the article and approved the submitted version.

## Funding

This work was supported in part by the program for Tianshan Innovative Research Team of Xinjiang Uygur Autonomous Region, China (2020D14020), and the Natural Science Foundation of China (11961071).

## Conflict of Interest

All the authors declare that the study was performed in the absence of any commercial or financial relationships that might be explicated as a potential conflict of interest.

## Publisher’s Note

All claims expressed in this article are solely those of the authors and do not necessarily represent those of their affiliated organizations, or those of the publisher, the editors and the reviewers. Any product that may be evaluated in this article, or claim that may be made by its manufacturer, is not guaranteed or endorsed by the publisher.

## References

[1] Garg SK Henry RR Banks P Buse JB Davies MJ Fulcher GR . Effects of Sotagliflozin Added to Insulin in Patients With Type 1 Diabetes. N Engl J Med (2017) 377(24):2337–48. doi: 10.1056/nejmoa1708337 28899222

[2] Chiang JL Kirkman MS Laffel LM Peters AL . Type 1 Diabetes Through the Life Span: A Position Statement of the American Diabetes Association. Diabetes Care (2014) 37(7):2034–54. doi: 10.2337/dc14-1140 PMC5865481 24935775

[3] El Masri D Ghosh S Jaber LA . Safety and Efficacy of Sodium-Glucose Cotransporter 2 (Sglt2) Inhibitors in Type 1 Diabetes: A Systematic Review and Meta-Analysis. Diabetes Res Clin Pract (2018) 137:83–92. doi: 10.1016/j.diabres.2018.01.004 29317332

[4] Bebu I Schade D Braffett B Kosiborod M Lopes-Virella M Soliman EZ . Risk Factors for First and Subsequent Cvd Events in Type 1 Diabetes: The Dcct/Edic Study. Diabetes Care (2020) 43(4):867–74. doi: 10.2337/dc19-2292 PMC7085803 32001614

[5] Frandsen CS Dejgaard TF Madsbad S . Non-Insulin Drugs to Treat Hyperglycaemia in Type 1 Diabetes Mellitus. Lancet Diabetes Endocrinol (2016) 4(9):766–80. doi: 10.1016/S2213-8587(16)00039-5 26969516

[6] Lebovitz HE . Insulin: Potential Negative Consequences of Early Routine Use in Patients With Type 2 Diabetes. Diabetes Care (2011) 34(Suppl 2):S225–30. doi: 10.2337/dc11-s225 PMC3632184 21525460

[7] Choudhary P Rickels MR Senior PA Vantyghem MC Maffi P Kay TW . Evidence-Informed Clinical Practice Recommendations for Treatment of Type 1 Diabetes Complicated by Problematic Hypoglycemia. Diabetes Care (2015) 38(6):1016–29. doi: 10.2337/dc15-0090 PMC4439532 25998294

[8] Tricco AC Ashoor HM Antony J Beyene J Veroniki AA Isaranuwatchai W . Safety, Effectiveness, and Cost Effectiveness of Long Acting Versus Intermediate Acting Insulin for Patients With Type 1 Diabetes: Systematic Review and Network Meta-Analysis. BMJ (2014) 349:g5459. doi: 10.1136/bmj.g5459 25274009 PMC4199252

[9] Russell-Jones D Khan R . Insulin-Associated Weight Gain in Diabetes–Causes, Effects and Coping Strategies. Diabetes Obes Metab (2007) 9(6):799–812. doi: 10.1111/j.1463-1326.2006.00686.x 17924864

[10] Chapman I Parker B Doran S Feinle-Bisset C Wishart J Strobel S . Effect of Pramlintide on Satiety and Food Intake in Obese Subjects and Subjects With Type 2 Diabetes. Diabetologia (2005) 48(5):838–48. doi: 10.1007/s00125-005-1732-4 15843914

[11] Frandsen CS Dejgaard TF Madsbad S Holst JJ . Non-Insulin Pharmacological Therapies for Treating Type 1 Diabetes. Expert Opin Pharmacother (2018) 19(9):947–60. doi: 10.1080/14656566.2018.1483339 29991320

[12] Herrmann K Frias JP Edelman SV Lutz K Shan K Chen S . Pramlintide Improved Measures of Glycemic Control and Body Weight in Patients With Type 1 Diabetes Mellitus Undergoing Continuous Subcutaneous Insulin Infusion Therapy. Postgrad Med (2013) 125(3):136–44. doi: 10.3810/pgm.2013.05.2635 23748514

[13] Kluger AY Tecson KM Barbin CM Lee AY Lerma EV Rosol ZP . Cardiorenal Outcomes in the Canvas, Declare-Timi 58, and Empa-Reg Outcome Trials: A Systematic Review. Rev Cardiovasc Med (2018) 19(2):41–9. doi: 10.31083/j.rcm.2018.02.907 31032602

[14] Jurczak MJ Lee HY Birkenfeld AL Jornayvaz FR Frederick DW Pongratz RL . Sglt2 Deletion Improves Glucose Homeostasis and Preserves Pancreatic Beta-Cell Function. Diabetes (2011) 60(3):890–8. doi: 10.2337/db10-1328 PMC3046850 21357472

[15] Dandona P Mathieu C Phillip M Hansen L Tschöpe D Thorén F . Efficacy and Safety of Dapagliflozin in Patients With Inadequately Controlled Type 1 Diabetes: The Depict-1 52-Week Study. Diabetes Care (2018) 41(12):2552–9. doi: 10.2337/dc18-1087 30352894

[16] Dandona P Mathieu C Phillip M Hansen L Griffen SC Tschöpe D . Efficacy and Safety of Dapagliflozin in Patients With Inadequately Controlled Type 1 Diabetes (Depict-1): 24 Week Results From a Multicentre, Double-Blind, Phase 3, Randomised Controlled Trial. Lancet Diabetes Endocrinol (2017) 5(11):864–76. doi: 10.1016/S2213-8587(17)30308-X 28919061

[17] Boeder S Edelman SV . Sodium-Glucose Co-Transporter Inhibitors as Adjunctive Treatment to Insulin in Type 1 Diabetes: A Review of Randomized Controlled Trials. Diabetes Obes Metab (2019) 21(Suppl 2):62–77. doi: 10.1111/dom.13749 31081593 PMC6899736

[18] Hutton B Salanti G Caldwell DM Chaimani A Schmid CH Cameron C . The Prisma Extension Statement for Reporting of Systematic Reviews Incorporating Network Meta-Analyses of Health Care Interventions: Checklist and Explanations. Ann Intern Med (2015) 162(11):777–84. doi: 10.7326/M14-2385 26030634

[19] Higgins JP Altman DG Gøtzsche PC Jüni P Moher D Oxman AD . The Cochrane Collaboration’s Tool for Assessing Risk of Bias in Randomised Trials. BMJ (2011) 343:d5928. doi: 10.1136/bmj.d5928 22008217 PMC3196245

[20] Atkins D Best D Briss PA Eccles M Falck-Ytter Y Flottorp S . Grading Quality of Evidence and Strength of Recommendations. BMJ (2004) 328(7454):1490. doi: 10.1136/bmj.328.7454.1490 15205295 PMC428525

[21] Liu Y Wang W Zhang AB Bai X Zhang S . Epley and Semont Maneuvers for Posterior Canal Benign Paroxysmal Positional Vertigo: A Network Meta-Analysis. Laryngoscope (2016) 126(4):951–5. doi: 10.1002/lary.25688 26403977

[22] Shim SR Kim SJ Lee J Rücker G . Network Meta-Analysis: Application and Practice Using R Software. Epidemiol Health (2019) 41:e2019013. doi: 10.4178/epih.e2019013 30999733 PMC6635665

[23] Caldwell D Ades A Higgins J. Simultaneous Comparison of Multiple Treatments: Combining Direct and Indirect Evidence. BMJ (2005) 331(7521):897–900. doi: 10.1136/bmj.331.7521.897 16223826 PMC1255806

[24] Lu G Ades AE . Combination of Direct and Indirect Evidence in Mixed Treatment Comparisons. Stat Med (2004) 23(20):3105–24. doi: 10.1002/sim.1875 15449338

[25] Dias S Welton NJ Caldwell DM Ades AE . Checking Consistency in Mixed Treatment Comparison Meta-Analysis. Stat Med (2010) 29:932–44. doi: 10.1002/sim.3767 20213715

[26] Salanti G Ades AE Ioannidis JP . Graphical Methods and Numerical Summaries for Presenting Results From Multiple-Treatment Meta-Analysis: An Overview and Tutorial. J Clin Epidemiol (2011) 64(2):163–71. doi: 10.1016/j.jclinepi.2010.03.016 20688472

[27] Mathieu C Dandona P Gillard P Senior P Hasslacher C Araki E . Efficacy and Safety of Dapagliflozin in Patients With Inadequately Controlled Type 1 Diabetes (the Depict-2 Study): 24-Week Results From a Randomized Controlled Trial. Diabetes Care (2018) 41(9):1938–46. doi: 10.2337/dc18-0623 30026335

[28] Mathieu C Dandona P Birkenfeld AL Hansen TK Iqbal N Xu J . Benefit/Risk Profile of Dapagliflozin 5 Mg in the Depict-1 and -2 Trials in Individuals With Type 1 Diabetes and Body Mass Index ≥27 Kg/M(2). Diabetes Obes Metab (2020) 22(11):2151–60. doi: 10.1111/dom.14144 PMC7693058 32691513

[29] Groop PH Dandona P Phillip M Gillard P Edelman S Jendle J . Effect of Dapagliflozin as an Adjunct to Insulin Over 52 Weeks in Individuals With Type 1 Diabetes: Post-Hoc Renal Analysis of the Depict Randomised Controlled Trials. Lancet Diabetes Endocrinol (2020) 8(10):845–54. doi: 10.1016/S2213-8587(20)30280-1 32946821

[30] Araki E Watada H Uchigata Y Tomonaga O Fujii H Ohashi H . Efficacy and Safety of Dapagliflozin in Japanese Patients With Inadequately Controlled Type 1 Diabetes (DEPICT-5): 52-Week Results From a Randomized, Open-Label, Phase III Clinical Trial. Diabetes Obes Metab (2020) 22(4):540–8. doi: 10.1111/dom.13922 PMC7078973 31742898

[31] Araki E Mathieu C Shiraiwa T Maeda H Ikeda H Thoren F . Long-Term (52-Week) Efficacy and Safety of Dapagliflozin as an Adjunct to Insulin Therapy in Japanese Patients With Type 1 Diabetes: Subgroup Analysis of the Depict-2 Study. Diabetes Obes Metab (2021) 23(7):1496–504. doi: 10.1111/dom.14362 PMC8251623 33620762

[32] Mathieu C Rudofsky G Phillip M Araki E Lind M Arya N . Long-Term Efficacy and Safety of Dapagliflozin in Patients With Inadequately Controlled Type 1 Diabetes (the Depict-2 Study): 52-Week Results From a Randomized Controlled Trial. Diabetes Obes Metab (2020) 22(9):1516–26. doi: 10.1111/dom.14060 PMC7496089 32311204

[33] Phillip M Mathieu C Lind M Araki E di Bartolo P Bergenstal R . Long-Term Efficacy and Safety of Dapagliflozin in Patients With Inadequately Controlled Type 1 Diabetes: Pooled 52-Week Outcomes From the Depict-1 and -2 Studies. Diabetes Obes Metab (2021) 23(2):549–60. doi: 10.1111/dom.14248 PMC7839492 33145944

[34] Mathieu C Dandona P Phillip M Oron T Lind M Hansen L . Glucose Variables in Type 1 Diabetes Studies With Dapagliflozin: Pooled Analysis of Continuous Glucose Monitoring Data From Depict-1 and -2. Diabetes Care (2019) 42(6):1081–7. doi: 10.2337/dc18-1983 30967434

[35] Mathieu C Dandona P Gillard P Senior P Hasslacher C Araki E . Efficacy and Safety of Dapagliflozin in Patients With Inadequately Controlled Type 1 Diabetes-Depict-2 Study. Diabetes (2018) 67(Supplement_1):213. doi: 10.2337/db18-213-OR

[36] Dandona P Mathieu C Phillip M Hansen L Tschoepe D Thoren F . Long-Term Efficacy and Safety of Dapagliflozin in Patients With Inadequately Controlled Type 1 Diabetes-The Depict-1 Study. Diabetes (2018) 67(Supplement_1):119–LB. doi: 10.2337/db18-119-LB 30352894

[37] Ludemann J Schaum T Mathieu C Xu J Thoren F . Pooled Analysis of the Duration of Type1 Diabetes in Dapagliflozin Vs Placebo on Adjustable Insulin Therapy From Depict 1 and 2: Effects on Glycaemia, Weight and Insulin Dosage. European Association for the Study of Diabetes (2018) 54(613).

[38] McKnight JA Wild SH Lamb MJ Cooper MN Jones TW Davis EA . Glycaemic Control of Type 1 Diabetes in Clinical Practice Early in the 21st Century: An International Comparison. Diabet Med (2015) 32(8):1036–50. doi: 10.1111/dme.12676 25510978

[39] Abu-Zaid A Altowairqi AK Dissanayaka T Oganesyan A Bhagavathul AS Alhabeeb H . A Systematic Review and Dose-Response Meta-Analysis on the Efficacy of Dapagliflozin in Patients With Type 1 Diabetes Mellitus. Pharmacol Res (2021) 165:105456. doi: 10.1016/j.phrs.2021.105456 33515709

[40] Chen J Fan F Wang JY Long Y Gao CL Stanton RC . The Efficacy and Safety of Sglt2 Inhibitors for Adjunctive Treatment of Type 1 Diabetes: A Systematic Review and Meta-Analysis. Sci Rep (2017) 7:44128. doi: 10.1038/srep44128 28276512 PMC5343472

[41] Yang Y Chen S Pan H Zou Y Wang B Wang G . Safety and Efficiency of Sglt2 Inhibitor Combining With Insulin in Subjects With Diabetes: Systematic Review and Meta-Analysis of Randomized Controlled Trials. Medicine (Baltimore) (2017) 96(21):e6944. doi: 10.1097/MD.0000000000006944 28538386 PMC5457866

[42] Yang Y Pan H Wang B Chen S Zhu H . Efficacy and Safety of Sglt2 Inhibitors in Patients With Type 1 Diabetes: A Meta-Analysis of Randomized Controlled Trials. Chin Med Sci J (2017) 32(1):22–7. doi: 10.24920/j1001-9242.2007.003 28399981

[43] Vallon V Thomson SC . Targeting Renal Glucose Reabsorption to Treat Hyperglycaemia: The Pleiotropic Effects of Sglt2 Inhibition. Diabetologia (2017) 60(2):215–25. doi: 10.1007/s00125-016-4157-3 PMC5884445 27878313

[44] Heerspink HJ Perkins BA Fitchett DH Husain M Cherney DZ . Sodium Glucose Cotransporter 2 Inhibitors in the Treatment of Diabetes Mellitus: Cardiovascular and Kidney Effects, Potential Mechanisms, and Clinical Applications. Circulation (2016) 134(10):752–72. doi: 10.1161/CIRCULATIONAHA.116.021887 27470878

[45] Li K Xu G . Safety and Efficacy of Sodium Glucose Co-Transporter 2 Inhibitors Combined With Insulin in Adults With Type 1 Diabetes: A Meta-Analysis of Randomized Controlled Trials. J Diabetes (2019) 11(8):645–55. doi: 10.1111/1753-0407.12890 30565398

[46] Yamada T Shojima N Noma H Yamauchi T Kadowaki T . Sodium-Glucose Co-Transporter-2 Inhibitors as Add-On Therapy to Insulin for Type 1 Diabetes Mellitus: Systematic Review and Meta-Analysis of Randomized Controlled Trials. Diabetes Obes Metab (2018) 20(7):1755–61. doi: 10.1111/dom.13260 29451721

[47] Watada H Shiramoto M Ueda S Tang W Asano M Thorén F . Pharmacokinetics and Pharmacodynamics of Dapagliflozin in Combination With Insulin in Japanese Patients With Type 1 Diabetes. Diabetes Obes Metab (2019) 21(4):876–82. doi: 10.1111/dom.13593 PMC6590304 30499157

[48] Danne T Garg S Peters AL Buse JB Mathieu C Pettus JH . International Consensus on Risk Management of Diabetic Ketoacidosis in Patients With Type 1 Diabetes Treated With Sodium-Glucose Cotransporter (Sglt) Inhibitors. Diabetes Care (2019) 42(6):1147–54. doi: 10.2337/dc18-2316 PMC6973545 30728224

[49] Ahmadieh H Ghazal N Azar ST . Role of Sodium Glucose Cotransporter-2 Inhibitors in Type I Diabetes Mellitus. Diabetes Metab Syndr Obes (2017) 10:161–7. doi: 10.2147/DMSO.S122767 PMC5422337 28496348

