# Supplementary material for: A Network Meta-Analysis of the Dose–Response Effects of Dapagliflozin on Efficacy and Safety in Adults With Type 1 Diabetes
Source: Front Endocrinol (Lausanne). 2022 Jul 7;13:923376. doi: 10.3389/fendo.2022.923376 (PMC9301373; doi:10.3389/fendo.2022.923376)
Supplement: Supplementary file 1 [file DataSheet_1.docx]

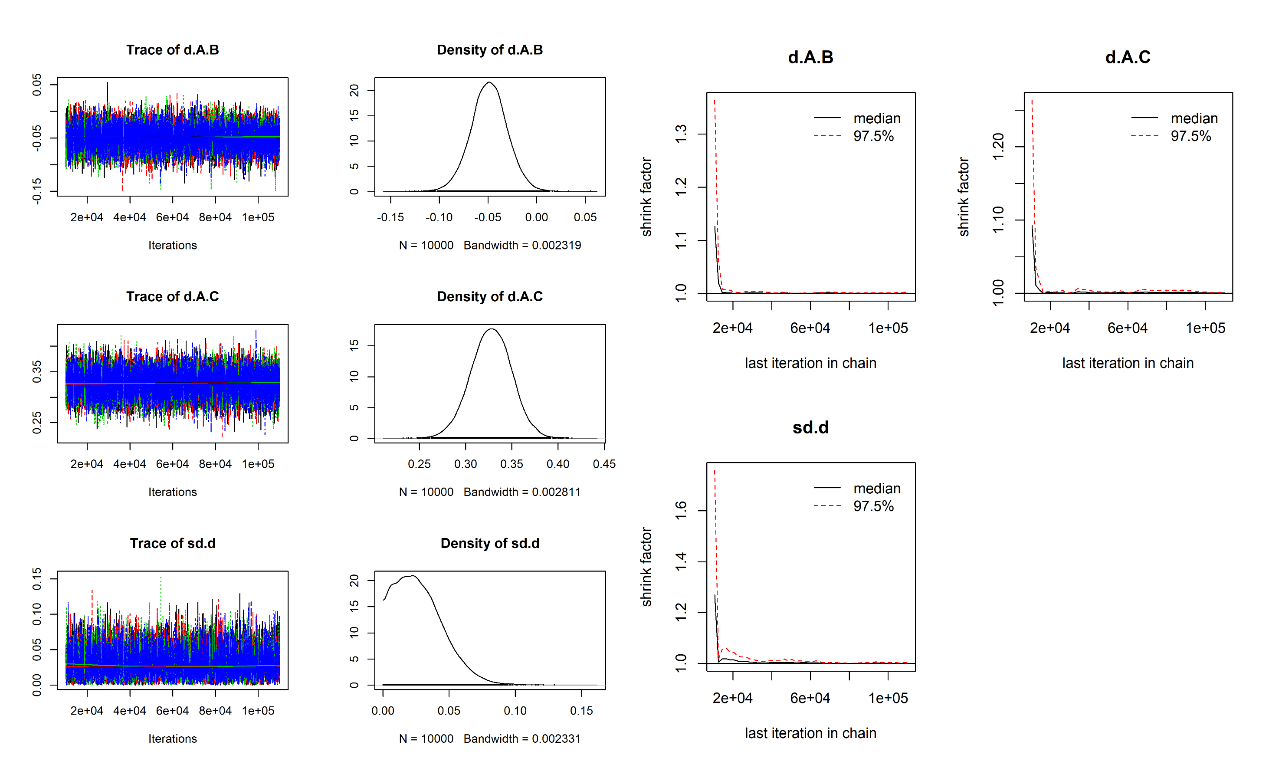


**FIGURE S1 |** The trace map, density map and convergent diagnostic diagram of HbA1c.


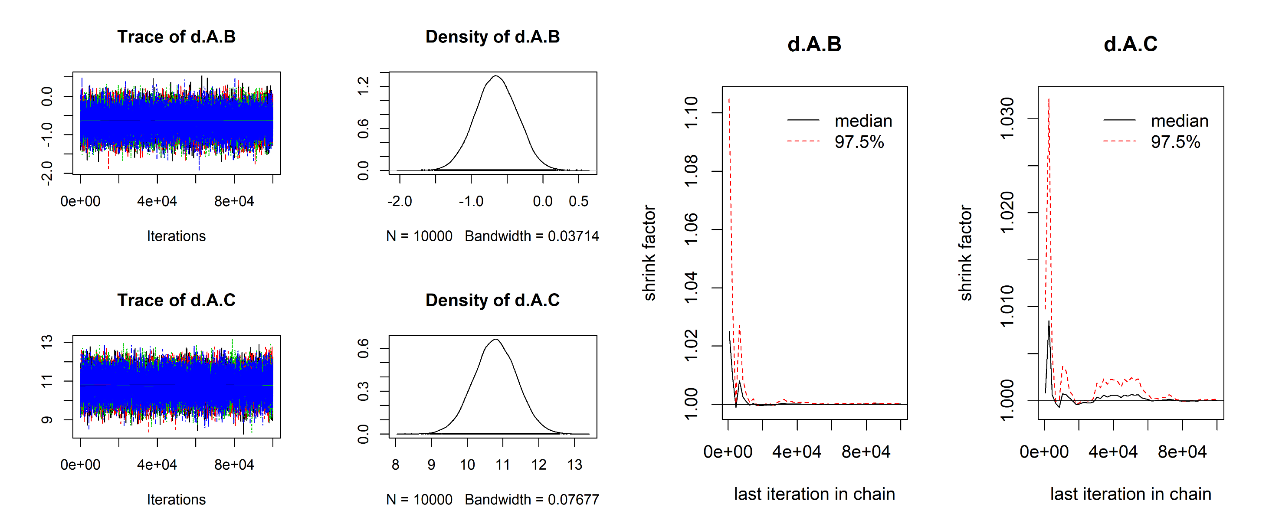


**FIGURE S2 |** The trace map, density map and convergent diagnostic diagram of TDD.


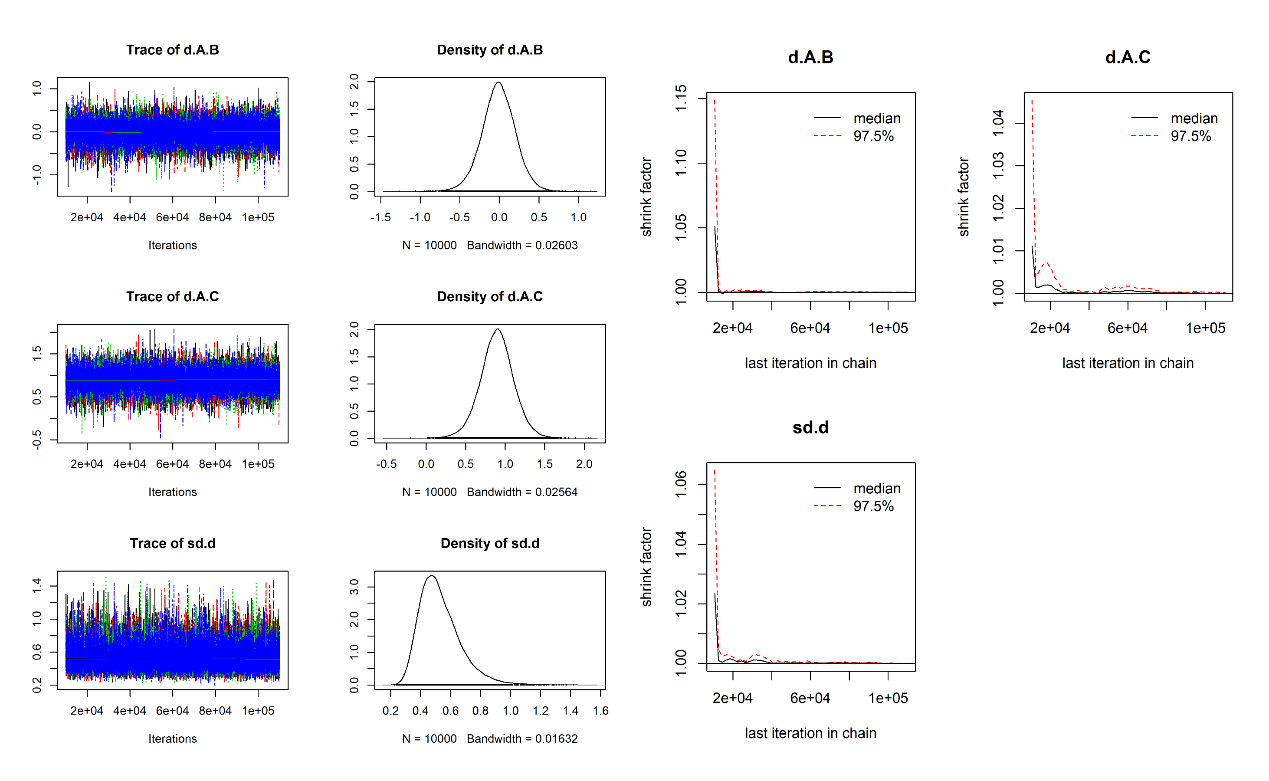


**FIGURE S3 |** The trace map, density map and convergent diagnostic diagram of 24h CGM.


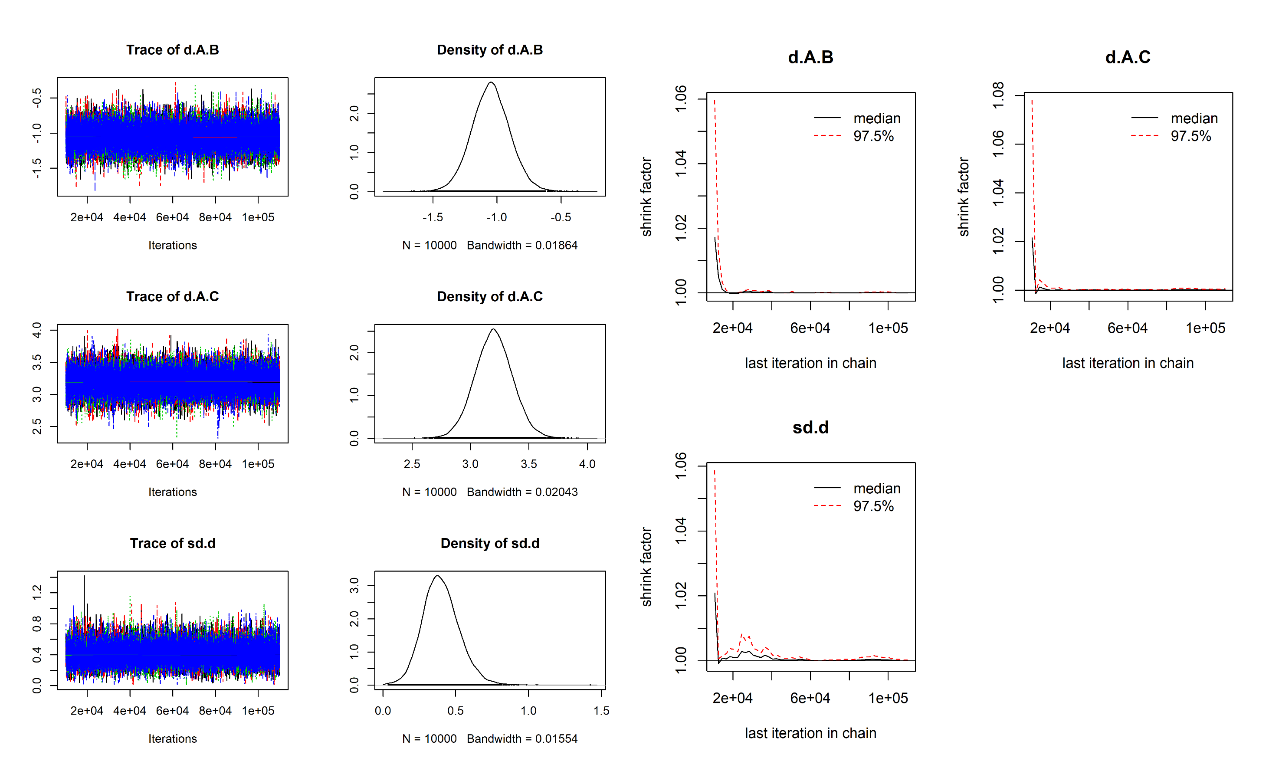


**FIGURE S4 |**The trace map, density map and convergent diagnostic diagram of body weight.


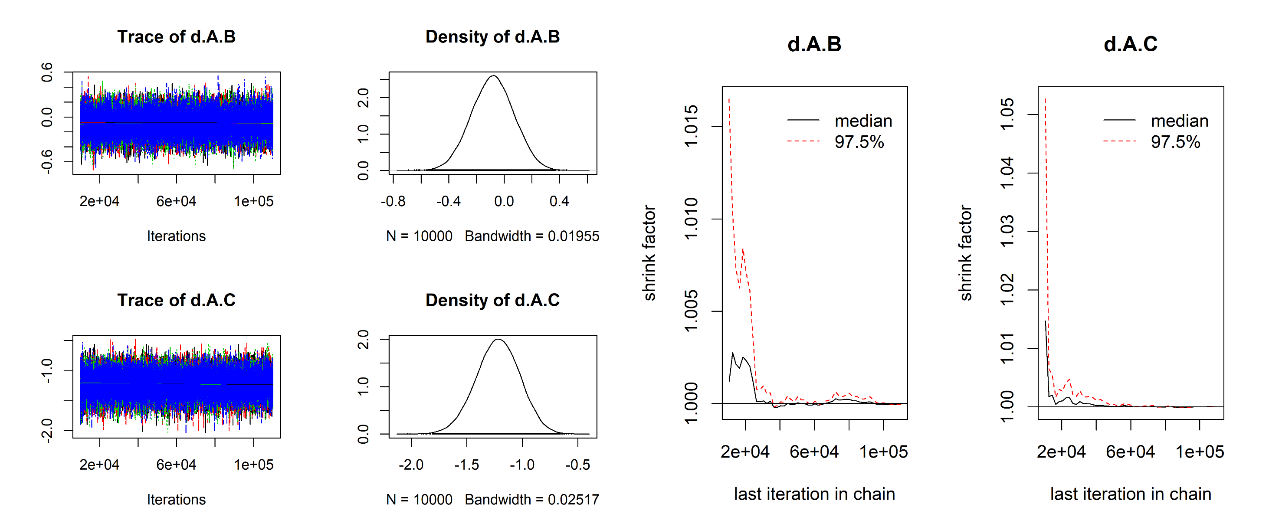


**FIGURE S5 |** The trace map, density map and convergent diagnostic diagram of DKA.


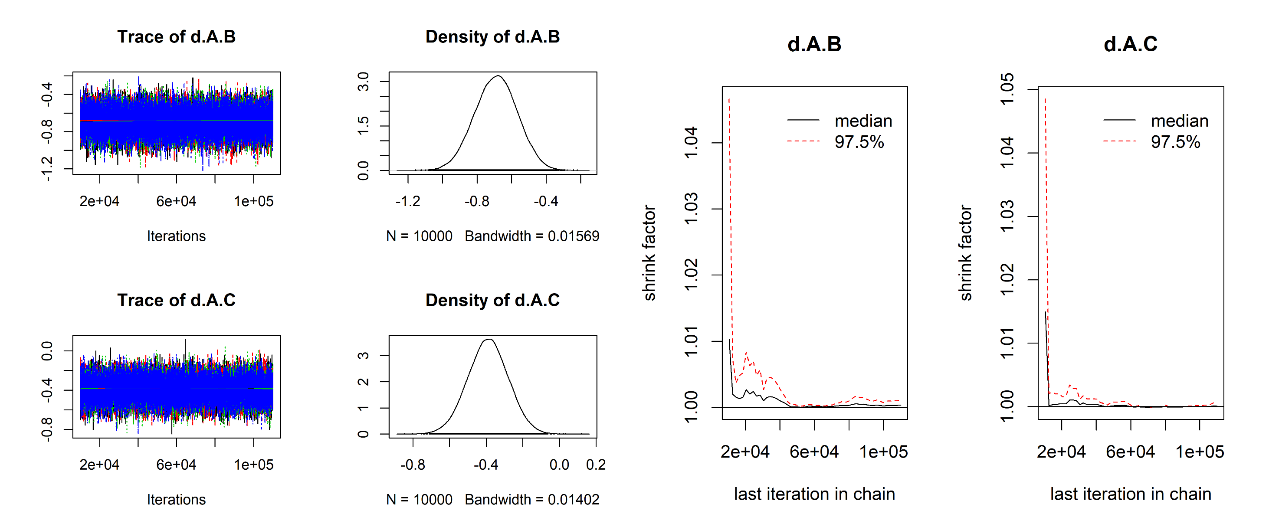


**FIGURE S6 |** The trace map, density map and convergent diagnostic diagram of urinary tract.


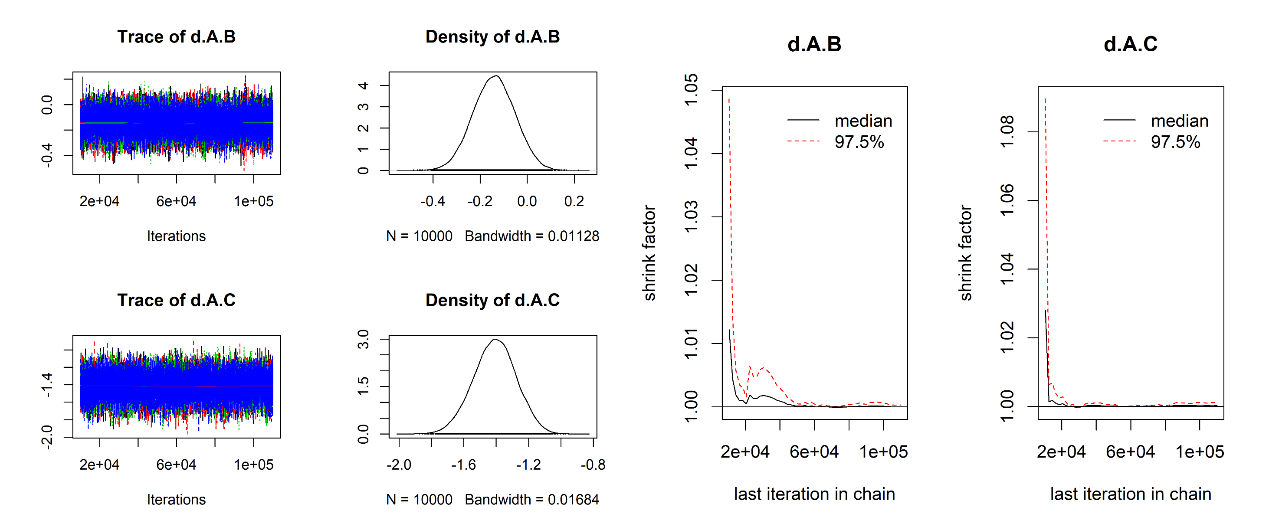


**FIGURE S7 |** The trace map, density map and convergent diagnostic diagram of genital infection.


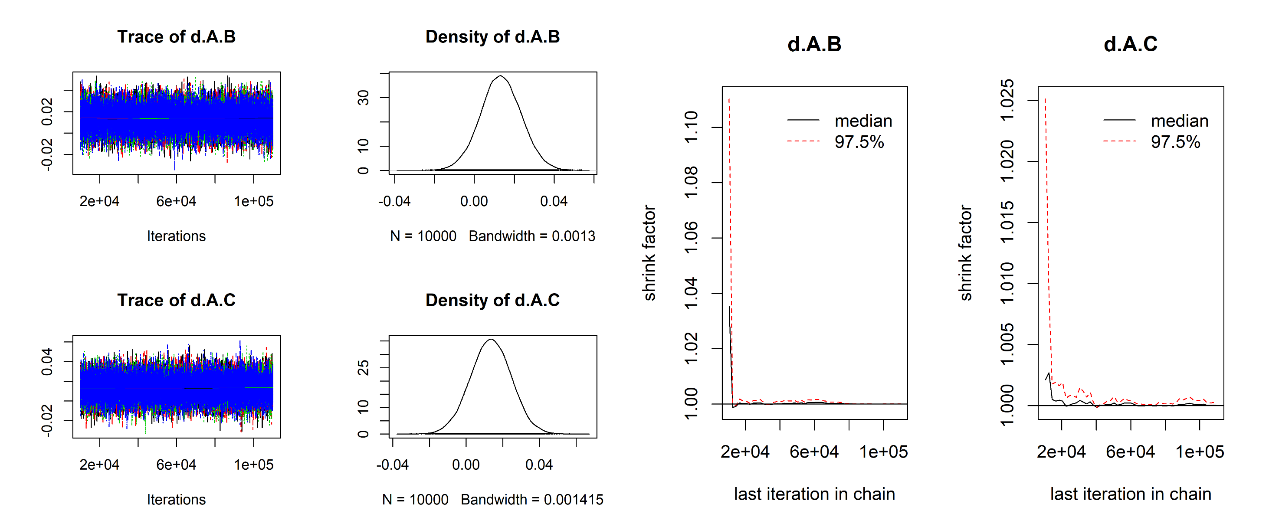


**FIGURE S8 |** The trace map, density map and convergent diagnostic diagram of hypoglycemia.


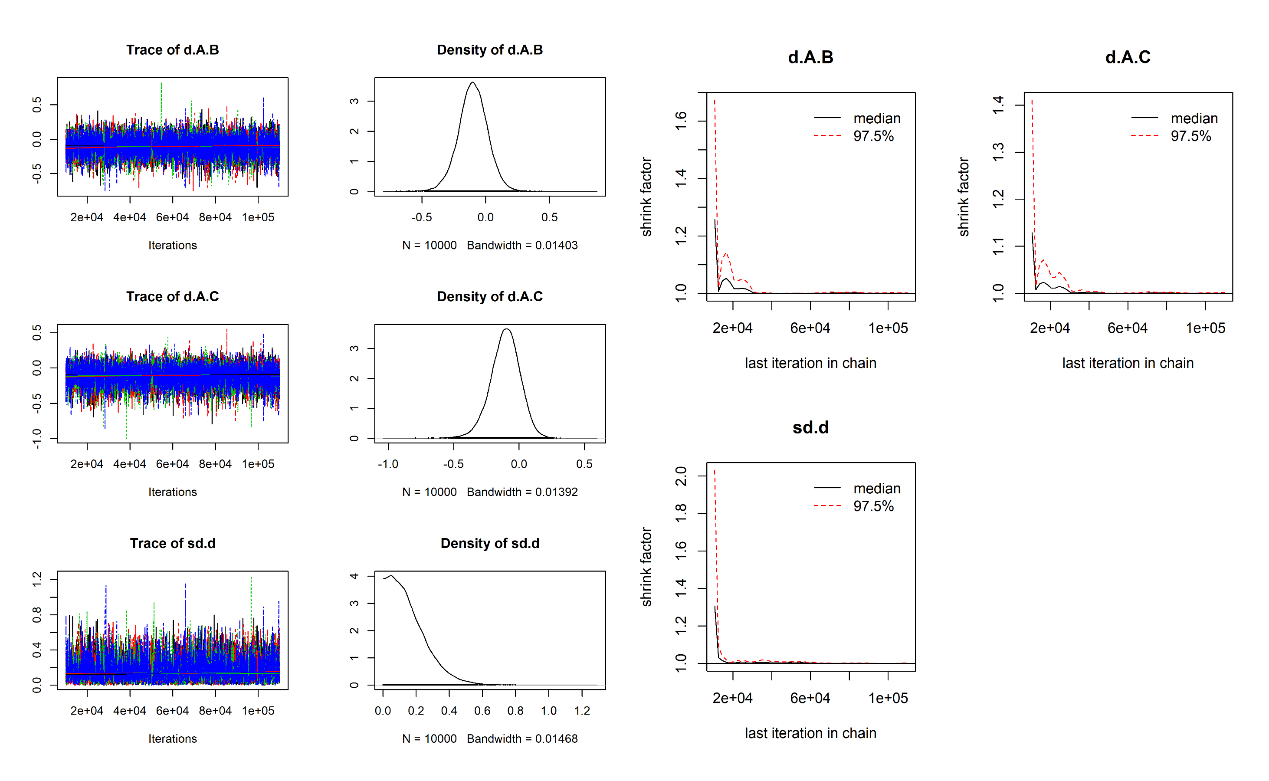


**FIGURE S9 |** The trace map, density map and convergent diagnostic diagram of severe hypoglycemia.
