# Supplementary material for: A Network Meta-Analysis of the Dose–Response Effects of Dapagliflozin on Efficacy and Safety in Adults With Type 1 Diabetes
Source: Front Endocrinol (Lausanne). 2022 Jul 7;13:923376. doi: 10.3389/fendo.2022.923376 (PMC9301373; doi:10.3389/fendo.2022.923376)
Supplement: Supplementary file 2 [file DataSheet_2.docx]

**Appendix 1: Search strategy for identifying RCTs assessing the effects of dapagliflozin combined with insulin in the treatment of patients with type 1 diabetes**

**PubMed**

((("Diabetes Mellitus, Type 1"[Mesh]) OR (((((((((((((((((((((((((((Diabetes Mellitus, Insulin-Dependent[Title/Abstract]) OR (Diabetes Mellitus, Insulin Dependent[Title/Abstract])) OR (Insulin-Dependent Diabetes Mellitus[Title/Abstract])) OR (Diabetes Mellitus, Juvenile-Onset[Title/Abstract])) OR (Diabetes Mellitus, Juvenile Onset[Title/Abstract])) OR (Juvenile-Onset Diabetes Mellitus[Title/Abstract])) OR (IDDM[Title/Abstract])) OR (Juvenile-Onset Diabetes[Title/Abstract])) OR (Diabetes, Juvenile-Onset[Title/Abstract])) OR (Juvenile Onset Diabetes[Title/Abstract])) OR (Diabetes Mellitus, Sudden-Onset[Title/Abstract])) OR (Diabetes Mellitus, Sudden Onset[Title/Abstract])) OR (Sudden-Onset Diabetes Mellitus[Title/Abstract])) OR (Type 1 Diabetes Mellitus[Title/Abstract])) OR (Diabetes Mellitus, Insulin-Dependent, 1[Title/Abstract])) OR (Insulin-Dependent Diabetes Mellitus 1[Title/Abstract])) OR (Insulin Dependent Diabetes Mellitus 1[Title/Abstract])) OR (Type 1 Diabetes[Title/Abstract])) OR (Diabetes, Type 1[Title/Abstract])) OR (Diabetes Mellitus, Type I[Title/Abstract])) OR (Diabetes, Autoimmune[Title/Abstract])) OR (Autoimmune Diabetes[Title/Abstract])) OR (Diabetes Mellitus, Brittle[Title/Abstract])) OR (Brittle Diabetes Mellitus[Title/Abstract])) OR (Diabetes Mellitus, Ketosis-Prone[Title/Abstract])) OR (Diabetes Mellitus, Ketosis Prone[Title/Abstract])) OR (Ketosis-Prone Diabetes Mellitus[Title/Abstract]))) AND (("dapagliflozin" [Supplementary Concept]) OR ((((((((2S,3R,4R,5S,6R)-2-(4-chloro-3-(4-ethoxybenzyl)phenyl)-6- (hydroxymethyl)tetrahydro-2H-pyran-3,4,5-triol[Title/Abstract]) OR (Farxiga[Title/Abstract])) OR (Forxiga[Title/Abstract])) OR (2-(3-(4-ethoxybenzyl)-4-chlorophenyl)-6-hydroxymethyltetrahydro-2H-pyran-3,4,5-triol[Title/Abstract])) OR (BMS 512148[Title/Abstract])) OR (BMS512148[Title/Abstract])) OR (BMS-512148[Title/Abstract])))) AND (randomized controlled trial[Publication Type] OR randomized[Title/Abstract] OR placebo[Title/Abstract])

| Table S1 \| Baseline characteristics of the included studies in the meta-analysis. | | | | | | | | | |  |
| --- | --- | --- | --- | --- | --- | --- | --- | --- | --- | --- |
| Study | **Number of patients(A/B/C)** | **Age Mean±SD (years)** | | |  | **Male/female (number)** | | | **Duration(weeks)** | |
|  |  | **A** | **B** | **C** |  | **A** | **B** | **C** |  |  |
| Mathieu C 2018 | 271/270/272 | 42.7±13.35 | 42.4±12.81 | 43±13.73 |  | 118/153 | 121/149 | 119/153 | 24 | |
| Mathieu C 2020 | 286/289 | 44.5±13.13 |  | 45±13.4 |  | 120/166 |  | 144/145 | 56 | |
| Groop PH 2020 | 80/84/76 | 42.9±12.9 | 45.6±13.7 | 44.9±11.9 |  | 41/39 | 44/40 | 52/35 | 52 | |
| Araki E 2020 | 76/75 | 47.7±12.9 | 48.9±12.9 |  |  | 43/33 | 33/42 |  | 52 | |
| Dandona P 2017^]^ | 259/259/260 | 41.9±14.1 | 42.7±14.1 | 42.7±13.6 |  | 111/148 | 130/129 | 132/128 | 24 | |
| Dandona P 2018 | 259/259/260 | 41.9±14.1 | 42.7±14.1 | 42.7±13.6 |  | 111/148 | 130/129 | 132/128 | 52 | |
| Araki E 2021 | 55/41/58 | 42.7 | 42.4 | 43 |  | 118/153 | 121/149 | 119/153 | 52 | |
| Mathieu C 2020 | 271/270/272 | 54.4±10.4 |  | 54.7±9.7 |  | 224/192 |  | 121/71 | 24 | |
| Phillip M 2020 | 548/566/532 | 42.4±13.7 | 42.9±13.4 | 42.9±13.6 |  | 237/311 | 273/293 | 251/281 | 52 | |
| Mathieu C 2019 | 530/529/532 | 42.3±13.7 | 42.6±13.4 | 42.9±13.6 |  | 229/301 | 251/278 | 251/281 | 24 | |
| MATHIEU C 2018 | 271/270/272 | NA | NA | NA |  | NA | NA | NA | 24 | |
| DANDONA P 2018 | 259/259/260 | NA | NA | NA |  | NA | NA | NA | 52 | |
| Ludemann J 2018 | 516/517/533 | NA | NA | NA |  | 253/263 | 236/281 | 234/299 | 24 | |

**NA: not available, A: dapagliflozin 5mg+insulin, B: dapagliflozin 10mg +insulin, C placebo+insulin.**

| Table S2 \| Results of network meta-analysis of HbA1c changes (%) | | |
| --- | --- | --- |
| dapagliflozin 5mg+insulin | -0.05 (-0.09, -0.01) | 0.33 (0.29,0.37) |
| 0.05 (0.01, 0.09) | **Dapagliflozin 10mg+insulin** | 0.38 (0.33,0.42) |
| -0.33 (-0.37, -0.28) | -0.38 (-0.42, -0.33) | **placebo+insulin** |

**The reported results are displayed with effect size and 95% confidence interval (95% CI). Mean difference (MD) is applied to continuous results.**

| Table S3 \| Results of network meta-analysis of percent change in total insulin daily dose (TDD) (%) | | |
| --- | --- | --- |
| dapagliflozin 5mg+insulin | -0.65 (-1.20, -0.07) | 11.00 (9.60,12.00) |
| 0.65 (0.07, 1.20) | **Dapagliflozin 10mg+insulin** | 11.00 (10.00,13.00) |
| -11.00 (-12.00, -9.60) | -11.00 (-13.00, -10.00) | **placebo+insulin** |
| The reported results are displayed with effect size and 95% confidence interval (95% CI). Mean difference (MD) is applied to continuous results.  Table S4 \| Results of network meta-analysis of change in 24h CGM | | |
| dapagliflozin 5mg+insulin | -0.01 (-0.45, 0.42) | 0.90 (0.47, 1.33) |
| 0.01 (-0.42, 0.45) | **Dapagliflozin 10mg+insulin** | 0.90 (0.48, 1.35) |
| -0.90 (-1.33 -0.47) | -0.90 (-1.35, -0.48) | **placebo+insulin** |

**The reported results are displayed with effect size and 95% confidence interval (95% CI). Mean difference (MD) is applied to continuous results.**

| Table S5 \| Results of network meta-analysis of percent change in body weight (%) | | |
| --- | --- | --- |
| dapagliflozin 5mg+insulin | -1.05 (-1.35, -0.75) | 3.20 (2.88,3.52) |
| 1.05 (0.75, 1.35) | **Dapagliflozin 10mg+insulin** | 4.25 (3.90,4.59) |
| -3.20 (-3.52, -2.88) | -4.25 (-4.59, -3.90) | **placebo+insulin** |

**The reported results are displayed with effect size and 95% confidence interval (95% CI). Mean difference (MD) is applied to continuous results.**

| Table S6 \| Results of network meta-analysis of rate of Diabetic ketoacidosis (DKA) | | |
| --- | --- | --- |
| dapagliflozin 5mg+insulin | 0.92 (0.68, 1.24) | 0.30 (0.20, 0.43) |
| 1.09 (0.81, 1.47) | **Dapagliflozin 10mg+insulin** | 0.32 (0.21, 0.48) |
| 3.38 (2.33, 5.05) | 3.11 (2.08, 4.76) | **placebo+insulin** |

**The reported results are displayed with effect size and 95% confidence interval (95% CI). Odds ratio (OR) is applied to binary results.**

| Table S7 \| Results of network meta-analysis of occurrence of urinary tract infection | | |
| --- | --- | --- |
| dapagliflozin 5mg+insulin | 0.50 (0.39, 0.64) | 0.68 (0.54, 0.85) |
| 1.99 (1.56, 2.59) | **Dapagliflozin 10mg+insulin** | 1.35 (1.04, 1.75) |
| 1.48 (1.17, 1.84) | 0.74 (0.57, 0.96) | **placebo+insulin** |

**The reported results are displayed with effect size and 95% confidence interval (95% CI). Odds ratio (OR) is applied to binary results.**

| Table S8 \| Results of network meta-analysis of risk of genital infection | | |
| --- | --- | --- |
| dapagliflozin 5mg+insulin | 0.88 (0.72, 1.07) | 0.23 (0.17, 0.30) |
| 1.14 (0.94, 1.39) | **Dapagliflozin 10mg+insulin** | 0.26 (0.19, 0.34) |
| 4.43 (3.38, 5.87) | 3.88 (2.93, 5.19) | **placebo+insulin** |

**The reported results are displayed with effect size and 95% confidence interval (95% CI). Odds ratio (OR) is applied to binary results.**

| Table S9 \| Results of network meta-analysis of risk of hypoglycemia | | |
| --- | --- | --- |
| dapagliflozin 5mg+insulin | 1.05 (0.92, 1.20) | 1.07 (0.93, 1.22) |
| 0.95 (0.83, 1.09) | **Dapagliflozin 10mg+insulin** | 1.02 (0.89, 1.17) |
| 0.94 (0.82, 1.07) | 0.98 (0.86, 1.13) | **placebo+insulin** |

**The reported results are displayed with effect size and 95% confidence interval (95% CI). Odds ratio (OR) is applied to binary results.**

| Table S10 \| Results of network meta-analysis of risk of severe hypoglycemia (%) | | |
| --- | --- | --- |
| dapagliflozin 5mg+insulin | 0.94 (0.72, 1.20) | 0.93 (0.71, 1.17) |
| 1.07 (0.83, 1.38) | **Dapagliflozin 10mg+insulin** | 0.99 (0.76, 1.27) |
| 1.08 (0.86, 1.40) | 1.01 (0.79, 1.32) | **placebo+insulin** |

**The reported results are displayed with effect size and 95% confidence interval (95% CI). Odds ratio (OR) is applied to binary results.**

| Table S11 \| Model fit statistics for all outcomes | | | | | | | |
| --- | --- | --- | --- | --- | --- | --- | --- |
| Outcome | **Model** | **DIC** | **Dbar** | **pD** | **ratio** | **I^2** | **Used in base case analyses** |
| HbA1c | FE | 54.24 | 38.18 | 16.06 | 1.005 | 3% | No |
|  | RE | 55.11 | 35.00 | 20.11 | 0.921 | 0% | Yes |
| TDD | FE | 32.33 | 20.33 | 11.99 | 0.782 | 0% | Yes |
|  | RE | 33.90 | 19.82 | 14.08 | 0.762 | 0% | No |
| 24h CGM | FE | 149.39 | 140.36 | 9.03 | 6.684 | 86% | No |
|  | RE | 41.27 | 21.30 | 19.98 | 1.014 | 6% | Yes |
| body weight | FE | 84.08 | 66.10 | 17.98 | 1.574 | 38% | No |
|  | RE | 73.47 | 43.04 | 30.44 | 1.025 | 5% | Yes |
| DKA | FE | 48.46 | 34.45 | 14.01 | 0.957 | 0% | Yes |
|  | RE | 50.16 | 34.73 | 15.43 | 0.965 | 0% | No |
| urinary tract | FE | 32.33 | 21.70 | 10.63 | 0.868 | 0% | Yes |
|  | RE | 34.22 | 22.39 | 11.83 | 0.896 | 0% | No |
| genital infection | FE | 23.39 | 13.67 | 9.71 | 0.621 | 0% | Yes |
|  | RE | 25.33 | 14.47 | 10.87 | 0.658 | 0% | No |
| hypoglycemia | FE | 34.17 | 20.99 | 13.18 | 0.618 | 0% | Yes |
|  | RE | 36.24 | 21.55 | 14.70 | 0.634 | 0% | No |
| severe hypoglycemia | FE | 50.51 | 39.03 | 11.48 | 1.346 | 28% | No |
|  | RE | 51.64 | 36.23 | 15.41 | 1.249 | 23% | Yes |

**DIC: deviance information criterion; FE, fixed effects; RE, random effects; HbA1c: glycated haemoglobin; TDD: total insulin daily dose; 24h CGM: 24 hour continuous glucose monitoring; DKA: diabetic ketoacidosis.**

| Table S12 *\|* Heterogeneity assessment in network | | | |
| --- | --- | --- | --- |
| outcomes | **numbers of trails** | **number of participants** | **heterogeneity(%)** |
| HbA1c | 14 | 8980 | 0 |
| TDD | 10 | 6351 | 0 |
| 24h CGM | 7 | 6702 | \ |
| body weight | 16 | 9051 | 29.54 |
| DKA | 13 | 10431 | 0 |
| urinary tract | 9 | 7561 | 0 |
| genital infection | 8 | 6728 | 0 |
| hypoglycemia | 12 | 9361 | 0 |
| severe hypoglycemia | 9 | 8387 | 0 |

**HbA1c: glycated haemoglobin; TDD: total insulin daily dose; 24h CGM: 24 hour continuous glucose monitoring; DKA: diabetic ketoacidosis; \: without.**

| Table S13 \| Assessment of local inconsistencies in different outcome indicators | | | | | | |
| --- | --- | --- | --- | --- | --- | --- |
| **Outcome indicators** | **Comparison** | **Direct WMD or OR (95%CI)** | **Indirect WMD or OR (95%CI)** | **Network**  **WMD or OR (95%CI)** | **P-value** |  |
| **HbA1c** | placebo+insulin vs dapagliflozin 10mg+insulin | 0.36  (0.30, 0.42) | 0.45  (0.30, 0.61) | 0.38  (0.33, 0.42) | 0.25 |  |
| **TDD** | placebo+insulin vs dapagliflozin 10mg+insulin | 1.5  ( -11.0, 14.0) | -9.1  (-19.0, 0.9) | -5.1  ( -13.0, 3.3) | 0.17 |  |
| **24h CGM** | / | / | / | / | / |  |
| **body weight** | placebo+insulin vs dapagliflozin 10mg+insulin | 4.10  (3.70, 4.40) | 5.00  (4.10, 5.90) | 4.25  (3.90,4.59) | 0.051 |  |
| **DKA** | placebo+insulin vs dapagliflozin 10mg+insulin | 0.31  (0.19, 0.47) | 0.74  (0.05, 22.00) | 0.32  (0.21, 0.48) | 0.515 |  |
| **urinary tract** | placebo+insulin vs dapagliflozin 10mg+insulin | 1.30  (0.98, 1.60) | 0.20  (0.01, 2.20) | 1.35  (1.04, 1.75) | 0.127 |  |
| **genital infection** | placebo+insulin vs dapagliflozin 10mg+insulin | 0.25  (0.19, 0.34) | 0.23  (0.02,2.30) | 0.26  (0.19, 0.34) | 0.909 |  |
| **hypoglycemia** | placebo+insulin vs dapagliflozin 10mg+insulin | 1.00  (0.98, 1.00) | 0.48  (0.10, 0.92) | 1.02  (0.89, 1.17) | **0.001** |  |
| **severe hypoglycemia** | \ | \ | \ | \ | \ |  |

**The reported results are displayed with effect size and 95% confidence interval (95% CI). Mean difference (MD) is applied to continuous results; Odds ratio (OR) is applied to binary results; HbA1c: glycated haemoglobin; TDD: total insulin daily dose; 24h CGM: 24 hour continuous glucose monitoring; DKA: diabetic ketoacidosis.**
